# Recent Advances and Perspectives of Carbon-Based Nanostructures as Anode Materials for Li-ion Batteries

**DOI:** 10.3390/ma12081229

**Published:** 2019-04-15

**Authors:** L. Selva Roselin, Ruey-Shin Juang, Chien-Te Hsieh, Suresh Sagadevan, Ahmad Umar, Rosilda Selvin, Hosameldin H. Hegazy

**Affiliations:** 1 Department of Chemistry, Faculty of Science and Arts, King Abdulaziz University, Rabigh, 21911 Rabigh, Saudi Arabia; 2 Department of Chemical and Materials Engineering, Chang Gung University, Guishan, Taoyuan 33302, Taiwan; 3 Division of Nephrology, Department of Internal Medicine, Chang Gung Memorial Hospital, Linkou-33305, Taiwan; 4 Department of Chemical Engineering and Materials Science, Yuan Ze University, Chungli, Taoyuan-32003, Taiwan; cthsieh@saturn.yzu.edu.tw; 5 Nanotechnology & Catalysis Research Centre, University of Malaya, Kuala Lumpur-50603, Malaysia; drsureshnano@gmail.com; 6 Department of Chemistry, Faculty of Science and Arts and Promising Centre for Sensors and Electronic Devices, Najran University, Najran 11001, Saudi Arabia; ahmadumar786@gmail.com; 7 Department of Chemistry, School of Science, Sandip University, Trimbak Road, Mahiravani, Nashik, Maharashtra 422213, India; selvinrosilda@yahoo.com; 8 Department of Physics, Faculty of Science, King Khalid University, Abha -61421, Saudi Arabia; hhegazy@kku.edu.sa; 9 Department of Physics, Faculty of Science, Al-Azhar University, Assiut 71524, Egypt

**Keywords:** Li-ion batteries, an anode, carbon-based nanomaterials

## Abstract

Rechargeable batteries are attractive power storage equipment for a broad diversity of applications. Lithium-ion (Li-ion) batteries are widely used the superior rechargeable battery in portable electronics. The increasing needs in portable electronic devices require improved Li-ion batteries with excellent results over many discharge-recharge cycles. One important approach to ensure the electrodes’ integrity is by increasing the storage capacity of cathode and anode materials. This could be achieved using nanoscale-sized electrode materials. In the article, we review the recent advances and perspectives of carbon nanomaterials as anode material for Lithium-ion battery applications. The first section of the review presents the general introduction, industrial use, and working principles of Li-ion batteries. It also demonstrates the advantages and disadvantages of nanomaterials and challenges to utilize nanomaterials for Li-ion battery applications. The second section of the review describes the utilization of various carbon-based nanomaterials as anode materials for Li-ion battery applications. The last section presents the conclusion and future directions.

## 1. Introduction

Environmentally friendly renewable energy sources have been explored for a sustainable future [1]. The present major energy sources are fossil fuel and nuclear energy. These nonrenewable energy sources have their own disadvantages. Fossil fuels are finite and will be depleted. Recent research has shown that fossil fuel energy sources emit greenhouse gases and carcinogenic substances [2]. Nonrenewable nuclear energy sources produce nuclear waste and are radioactive. Now, there is evidence of global warming and researchers from many fields are focusing on alternative energy sources; e.g., fuel cells, photovoltaic cells, and photoelectrochemical cells [3]. The energy produced from the green techniques requires storage devices. An electrochemical device such as a battery can store electricity inside a closed energy system as chemical energy and can be recharged and re-used as a power source in small electrical appliances, large machinery, and inaccessible locations [4]. Electrical energy is obtained by an electrochemical redox chemical reaction. Many electronic types of equipment cannot work without batteries [5]. There is increased usage of portable electronic systems of personal use. These developments have resulted in a massive demand for batteries [6]. In the near future, all electronic devices will be turned into portable and flexible ones. In addition, the use of rechargeable battery technology has many benefits for armed forces, such as in missiles, ships, submarines and aircraft, satellites, space stations, etc. In industry, where handheld devices are replacing clipboards and paperwork, and it provides higher levels of accuracy in tests once performed by hand [7]. Therefore, various electronic devices are driving the battery market to greater growth than ever before, especially the growth of rechargeable batteries.

The chemical unit inside a battery is called a cell. Many cells connected in series to form one battery. Every cell has three important portions; a positive electrode, a negative electrode, and a liquid or solid electrolyte [8]. While charging the battery with electric current, a chemical reaction happens in the electrolyte, making ions move through it one way, with electrons moving through the external circuit the other way [9]. This development of electric charge makes an electric current flow through the cell [10].

Although there are different kinds of batteries available, based on their working function they are classified into two types: primary (disposable) and secondary (non-disposable) batteries [11]. Primary cells, for the most part, have a better shelf life and are simple to use, but they cannot be recharged and discarded. These primary batteries are simple and helpful versatile power sources for different applications including medical devices, defense components, and so on [12]. Zinc-carbon batteries and alkaline batteries are a sort of disposable batteries [13]. Alkaline Zn–Mn batteries have assumed control a large portion of the current primary battery market, and their generation and utilization keep on developing in light of their invaluable expense and execution [14]. Generally, the energy densities of these alkaline Zn–Mn batteries are higher than the secondary batteries. The secondary batteries are designed for recharging and reuse. The benefits achieved by secondary cells over primary cells are their reusability, higher power density, and higher discharge rate with excellent performance at low-temperature [15]. Various kinds of commercial battery systems are available in the market, including lead-acid battery, nickel–cadmium, nickel–metal hydride, nickel–zinc, zinc–air, zinc–bromine, and lithium batteries. Jürgen Garche discussed the characteristics of these battery systems in detail [16]. Much advancement has additionally been attempted in creating essential metal–air and lithium batteries, which utilize light metals as the anode. Lithium is a light metal and may convey a theoretical limit up to 3860 mAh/g. Since lithium plating may cause hampering in batteries, magnesium is an elective lightweight component with lower cost, lower toxicity, and higher security [17]. Likewise, Mg is rich in both the earth’s crust and seawater. Correspondingly, other active metals incorporate aluminum and zinc are alluring electrode materials with a high limit (theoretically 2980 and 820 mAh/g for Al and Zn, respectively). In any case, the practical capacity of the utilized active materials for primary batteries remains are restricted because of the low use efficiency of the conventional bulk form [18].

Lithium is the first choice for negative electrode material as it is the lightest, most electropositive metal with high power density energy. Theoretically, the specific capacity for lithium is higher than zinc and lead [19]. The specific capacity values for lithium, zinc, and lead are 3860, 820, and 260 Ah/kg, respectively [20]. In 1949, Hajek first suggested the use of lithium in a battery. In the 1960s, research on lithium batteries began and the concept of “lithium secondary batteries” was presented [21]. At the beginning of the 1980s, the researchers showed interest in secondary lithium batteries that reversibly incorporate lithium ions in their structure. Using lithium as the anode created a series of problems. Corrosion of lithium occurred, which led to dendrite formation and thus a high-volume change of the anode during cycling that resulted in a low cycling efficiency of less than 200 cycle life [22]. More importantly, the highly reactive lithium raised mainly safety concerns about the type of cell. The high reactivity of lithium made an electrolyte impossible. Two methodologies were proposed later in 1980 to overcome this issue. The main methodology comprised of utilizing a strong polymer electrolyte, which is less reactive with lithium; prompting all-solid-state lithium metal rechargeable batteries. The second conceivable approach to remove the issue related with Li metal is to supplant it with material ready to intercalate lithium ions reversibly at a low voltage, prompting the so-called “lithium-ion”, “rocking chair”, or “swing” lithium rechargeable batteries [23]. In the first approach, it was necessary to find a suitable electrolyte medium and a reversible positive electrode material to avoid lithium reacting with the electrolyte. Therefore, a nonaqueous slid electrolyte is needed for the lithium metal anode. LiCl–AlCl_3_ electrolyte mixed and dissolved with propylene carbonate was proposed by Chilton and Cook [21]. As for the cathode, different halides, such as AgCl, CuF_2_, CuCl, CuCl_2_, and NiF_2_, were examined initially in the prototype cells. During discharging, these halides are converted into lithium halide and their corresponding metal. The formation of soluble complexes of the cathode leads to a high self-discharge rate. The alloys of lithium with other metals such as Al were studied to substitute the Li metal [24].

### 1.1. Industrial Use of Lithium-Ion Battery Technology

Sony Energetic of Japan utilized the Li-ion secondary battery for the first time. Li-ion batteries have many advantages over other rechargeable batteries [25]. First, they have a higher output voltage; the voltage of a single cell is ~4.1 V, approximately three times higher than that of NiCd and NiMH cells and two times higher than that of lead–acid batteries. Because of the higher voltage, less lithium-ion cells are required in a battery of a given voltage [26]. Also, higher energy density results in smaller and lighter batteries. Different advantages are low self-discharge, (2–10% every month, amazing cycle life (more noteworthy than 1000 cycles) wide employed temperature extend (−20 to 60 °C), long time spans of usability (up to five years), and low upkeep [18]. The cells do not show memory impact (where utilization time is condensed by repeated shallow discharge cycles) which is a component of NiCd batteries [27]. The gravimetric vitality thickness (~150 Wh/kg) of lithium-particle cells is twice that of NiMH cells and the volumetric energy density (~400 Wh/L) is four times more noteworthy than that of NiCd cells [28]. Moreover, lithium-ion cells give high rate ability: 5 C persistent discharge rates and 25 C consistent discharge rates have been reported in the literature [29]. These properties make lithium-ion technology appealing to different applications. Hardly any disadvantages observed incorporate moderately high costs, long charging times, capacity loss, and poor cyclability. These issues will, in general, begin from the electrode-electrolyte interfaces. 

### 1.2. Working Principles of Li-Ion Batteries

A battery is a device that changes chemical energy into electric energy by means of electrochemical oxidation-reduction reaction (redox) reaction. The essential electrochemical unit achieving such energy change is known as a “cell” [30]. A battery contains a group of interconnected cells. The number of cells utilized relies upon the desired capacity and voltage for a specific application. A few electrochemical cells are associated with the arrangement as well as in parallel to acquire a lithium-ion battery of indicated voltage and capacity [31]. Each cell contains the following parts: a negative terminal (anode), where electrochemical oxidation happens during discharge; a positive terminal (cathode), where electrochemical decrease happens; an electrolyte, which encourages the transportations of ions from one electrode to another electrode; a separator, which gives electronic segregation between the electrodes; and a casing, which contains the other cell parts [32]. Lithium-ion cells use a solid reductant as an anode and a solid oxidant as a cathode. The cathode materials used in most of the commercial Li-ion batteries are LiCoO_2_ or LiNiO_2_ and the anode materials are carbonaceous [33]. During cell charging, the cathode material releases Li ions to the electrolyte and electrons are removed from the cathode by applying an external field and are then transferred to the anode. The charge-compensating Li ions are attracted by the negative electrode and then inserted into it. During cell discharge, the reverse reaction occurs [8]. That is, the anode supplies intercalated Li ions into the electrolyte and provides electrons to the external circuit. At the cathode, the Li ions intercalate from the electrolyte and satisfy the charge of electrons from an external circuit [34]. Common carbon anode materials are graphite or coke-type or both combined; common cathode materials include LiMn_2_O_4_, LiCoO_2_, and LiNiO_2_. The electrolyte can be either solid or liquid. The liquid electrolyte is usually a nonaqueous solution of Li salts and various solvents including ethers, esters, and carbonates [35]. The cell reactions that occur at the cathode and anode during cell charging and discharging are represented as follows (Equations (1)–(4)) [36,37,38]: 

during charging

At cathode: LiMO_2_ → Li_1−x_MO_2_ + xLi^+^ + xe^−^ (1)

At anode: xLi^+^ + xe^−^ + C → Li_x_C (2)

during discharging

At cathode: Li_1−x_MO_2_ + xLi^+^ + xe^−^ → LiMO_2_ (3)

At anode: Li_x_C→ xLi^+^ + xe^−^ + C (4)

Connecting the electrodes to an external load results in electron flow in the external circuit and the ions move through the electrolyte [39]. The charge flows result from electrochemical (or redox) reactions at the electrodes that include chemical species and electrons and occur at various voltages [40]. The output voltage of the battery is given by the voltage difference between the two redox reactions. In a primary battery, the redox reactions can’t be turned around and the system must be discharged once, while in a secondary (or rechargeable) battery, the redox reactions are reversible, which results in multiple charging and discharging cycles [41]. Energy storage and conversion materials with lightweight active materials involving H^+^ and OH^−^ ionic transport in an electrochemical reaction are being developed which facilitate miniature electronic devices. However, most conventional batteries have a lower kinetic rate of ion diffusion and migration [42]. This leads to low electrode performance compared to the theoretical value. Hence, scientists and engineers are involved in developing new electrode materials to improve the battery performance of current batteries. Current research on battery technologies is focused on (i) developing new anode/cathode materials with high-energy storage capacity and cyclability, (ii) investigation of liquid-free solid electrolyte materials with higher ionic conductivities, which addresses the flammability of organic solvents, and (iii) optimization of the electrode-electrolyte interface to improve cell safety and lifetime. Apart from these, simple battery fabrication methods utilizing thin, lightweight, and cost-effective materials are sought for next-generation batteries [43].

### 1.3. Utilization of Nanomaterials for Li-Ion Batteries

To improve the electrode performance of a battery, it is necessary to improve the (i) specific charge and charge density, cell voltage, and reversibility of cathode and anode electrochemical reactions [44]. Improvisation in lithium batteries can be done by using different materials for the cathode, anode, and electrolyte. In general, the electrochemical properties of electrode materials depend on crystallinity, phase purity, particle size and grain size of materials, and particle size distribution [45]. Both the size of the particles and size of grains within these particles play important roles, in which electrodes having smaller particles and grains show better cyclability. The power density of the microsized particle is relatively low due of a high polarization at a high charge-discharge rates [46] and is caused by moderate diffusion in the active material and rises in the resistance of the electrolyte. This issue can be resolved by planning active nanostructure materials that give high surface area and short diffusion ways for ionic transport and electronic conduction. Therefore, the use of nanoparticles as active materials dispersed in a lithium oxide or lithium salt matrix could decrease the diffusion path length for lithium insertion and decreases the charge transfer resistance of the electrode, thereby increasing the rate (power) capability of the battery [47]. Nanoparticles prevent aggregation and formation of large particles (thus avoiding the cracks). It has been confirmed in many studies that the cycling performance of alloy anodes can be improved by reducing the active particle size to the nanometer range (<100 nm) and by circumventing agglomeration of the particles by composite electrode materials [48]. The improved cycling stability can be attributed primarily to nanosized particles to accommodate large stress and strain without any cracking. 

### 1.4. Advantages of Nanomaterials for Li-Ion Batteries

Micrometer-sized electrode materials are mostly used in commercial batteries, which are limited by their kinetics, lithium-ion intercalation capacities, and structural stability, resulting in low specific energy and energy density with low reversibility of electrochemical reactions. Many benefits are observed for lithium ion batteries when using nanomaterials: 

(i) Since the nanostructured materials provide smaller diffusion length and higher surface/volume ratio than bulk materials, the diffusion of Li^+^ ions are faster and thus the rate of charging is faster. The electrochemical redox reaction in Li-ion batteries proceeded in three stages; diffusion of the Li-ion inside the electrode materials, a charge transfer effect between the boundary of the electrode and the electrolyte, and Li-ion progress in the electrolyte [31]. Amongst these, the diffusion of Li ions is generally considered to be the rate-determining step of Li-ion batteries. Literature revealed that the diffusion kinetics could be improved by two approaches [49]. In one approach, doped electrode materials are used [50]. Nevertheless, the introduction of impurities sometimes causes an unstable crystal lattice which leads to lesser improvement in rate-performance. The second approach involves utilizing nanostructured materials which could minimize the diffusion length of Li^+^ and e^−^ [36]. The use of nanomaterials significantly shortens the Li+ and e^-^ diffusion lengths to the nanometer scale within the electrode materials and hence increases the rate (power) capability of the battery. 

(ii) Li surface storage due to nanosize may play an important role in the overall capacity. The use of nanomaterials enhances the electrode/electrolyte contact area resulting from the larger surface area thereby enhances the electrode capability of Li storage [51]. The storage of lithium could be possible at its lattices, surface, interface, and in its nanopores. In addition to the large surface area, enhanced electrode capacity resulted in a new Li-ion storage mechanism [52]. The classical mechanism involves either a Li insertion/de-insertion process or a Li alloy formation. To understand the role of nanoparticles in the mechanism of Li reactivity, Poizot et al. [37] constructed LiMn_2_O_4_/CoO cells and studied the structural properties during charging and discharging. The CoO materials form a rock-salt structure which does not occupy Li-ions for insertion [53]. Also, it does not form an alloy with Li. It was observed that during charging and discharging, there were a reversible formation and decomposition of Li_2_O. This new type of mechanism is called as ‘conversion mechanism’ (Equation (5)).

MX + yLi^+^ + ye^−^ ↔ LiX + M (5)

In the case of Cu_3_N as a negative electrode, a conversion mechanism is likely to happen, wherein Cu metal is oxidized into Cu^2+^ to form CuO and its reduction contributes to the increase in capacity with cycle number. The additional Li storage capacity in nanosize transition metal oxide at low potential could also be explained by another mechanism, called the interfacial Li storage mechanism [38]. As per this concept, Li^+^ ions are stored in the subsurface or surface of Li_2_O or LiF, while the electrons are suited in the transition metal, resulting in charge separation. In nanostructured solids, such interfacial effects show the dominant role because of their significant ratio of interfaces with respect to the bulk [54]. It is significant to take note that interfacial storage is seen in the Me/LiX, X=O, F nanocomposites, in which no compound reactions occur between the transition metal and Li. The two parts, Me and LiX network, can’t store Li in their bulk. It is the synergistic two-phase effect that allows additional storage at low potential [55]. This concept highlights the significance of synergistic attentions in composite systems and it forms the bridge between the capacitor and electrode function [56]. For example, in the case of LiCoO_2_, as the crystallite size decreased from 17 to 8 nm, the capacity contribution increased from 2.6 to 6.4 mAh/g. Interestingly, the charging (deintercalation) potentials decreased with size. The lower potentials suggest the effect of nanosize on the electrochemical performance of LiCoO_2_ [57]. 

(iii) In conventional bulk materials, there is considerable volume change occur during the Li insertion and de-insertion process, which causes cracking in the anode that leads to the capacity loss in batteries. It is believed that nanosized anode materials can overcome the volume change during the Li insertion and de-insertion process and so the stress and strain could be surmounted. This problem is mostly observed in silicon anodes. The theoretical capacity of silicon anode is 4200 mAh g^−1^, which is ten times higher than that of graphite anodes. However, there is a huge amount of capacity loss was observed in these silicon anodes. This is due to the higher number of Li atoms (4.4 Li atoms) could be inserted for one Si atom during Li insertion, which leads to higher volume expansion. On the other hand, during the Li de-insertion process, huge volume contraction is observed. This type of volume change causes cracking of Si anodes. The loss in electrical contact leads to capacity loss. When the particle size of anode materials is small, the volume of the anode is maintained, and the capacity loss can be avoided. Comparison of nanoscale single crystalline SnO and conventional bulky SnO electrode showed that the nanoscale single crystalline SnO exhibited higher specific capacity with better cycle performance [58]. In the SnO meshed plate nanostructure with nanosized ribbons, the nanoribbons provide a space to maintain the volume extension. Similarly, in layered Li_x_Mn_1-y_Co_y_O_2_ cathodes, spinel-like phases are formed, with every crystallite being made of a mosaic of nanodomains. The strain is overcome at the domain wall boundaries, leading to facile cycling of these nanostructured materials compared to the spinels obtained at high-temperature without nanodomains [59].

(iv) Electrochemical reactions of the irreversible bulk materials can be made reversible when it comes to the nanoscale. These results with higher specific capacity than compared to that of the theoretical value. For example, in tin-based Li storage compounds, for example, tin oxide, it is irreversibly converted to tin according to the equation given below (Equations (6) and (7)). The second reaction involves the formation of several phases in the LixSn system, which is reversible.

SnO_2_ + 4Li^+^ + 4e^−^ → Sn + 2Li_2_O (6)

Sn + xLi^+^ + xe^−^↔Li_x_Sn ((0 ≤ x ≤ 4.4) (7)

when pure SnO_2_ nanomaterial was used, the irreversible first reaction (Equation (6)) could be more reversible, resulting in higher specific capacity than the theoretical value. 

(v) Nanotubes and nanowires can enhance electrical percolation and mechanical properties by entanglement [60].

**Figure 1:**
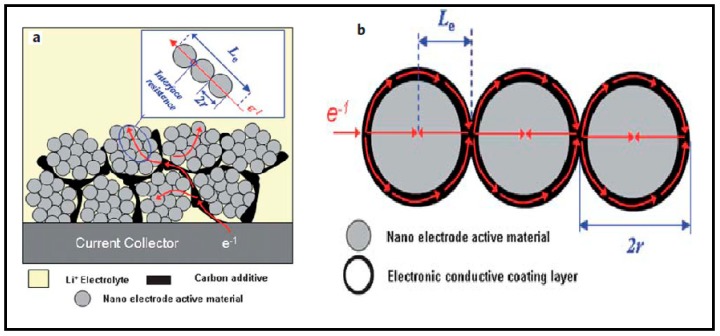
(**a**) Schematic representation showing the electronic transport length (L_e_) in the nanoparticles-based electrode (**b**) Schematic illustration of the electronic transport length for nanoparticles with a full conductive coating [63].

Nanomaterials with a high surface-to-volume ratio increase the area of contact between the electrolyte and the electrode, resulting in more exposed redox sites providing higher power and energy density. The mechanically strong, low-dimensional nanomaterials offer high resistance to any structural damage. In addition, their structure can be easily tuned to overcome changes in the volume of lithium-based battery electrodes. Nanomaterials offer new lithium storage mechanisms irrespective of the simple ion accumulation at insertion sites, such as surface redox reactions, lithium storage at interfaces, and nanopores inherently present in nanostructures. Most electrode materials have successfully applied the strategy that enhances the rate capability with nanosized particles. Combining the advantage of low cost, low toxicity, and the ability to be manufactured as a nanomaterial that delivers fast lithium-ion intercalation, a great deal of attention has recently been given to titanium oxides [61,62]. However, “nanosize” can only effectively shorten the path length for electronic transport in practical application. In Figure 1a, where a carbon additive is used to improve the conductivity of the total electrode, the electronic transport in a nanomaterial-based electrode is shown schematically. Since nanosized particles have a very high specific surface area and high surface energy and tend to form agglomerates, they are hard to disperse and are mixed with a carton additive (see Figure 1a) [63]. The electronic transport length (Le) is therefore still much higher than the particle size (r) because only a small amount of nanoparticles can contact the carbon additive directly and obtain electrons (see Figure 1b). In addition, there is still a large interface resistance, particularly if the particle size is within a typical nanoscale [63]. If each nanoparticle is fully covered with an electronic conductive layer, it would effectively shorten the electronic transport length (Le) in an electrode. In this case, the electronic transport length (Le) throughout the active material is equal to (or less than) the nanomaterial particle size (r) (as shown in Figure 1b). This is because every nanoparticle’s outer surface can be completely passed by electrons. Being nanosized can effectively reduce the electronic transport length (Le) across the active material with a thin electronic conductive coating layer. In addition, the electronic conductive layer of coating can reduce the resistance of the interface.

### 1.5. Disadvantages of Nanomaterials for Li-Ion Batteries

**Figure 2:**
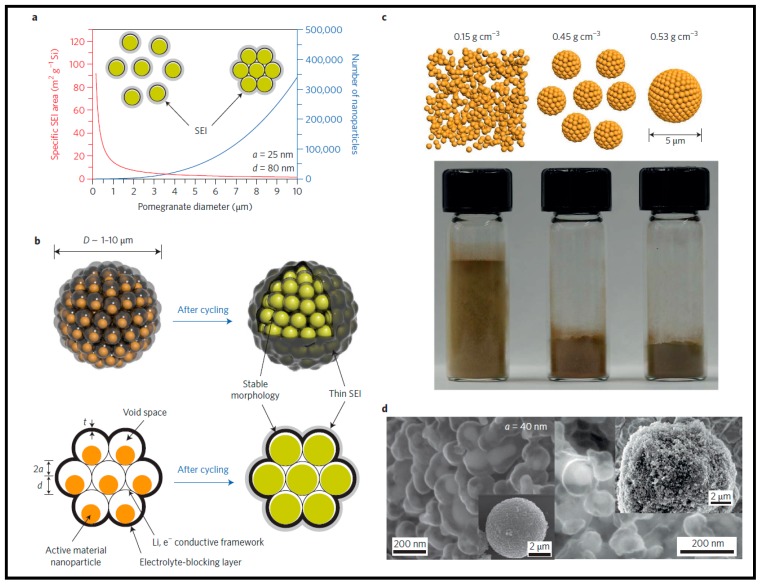
(**a**) Calculated surface area in contact with the electrolyte (specific SEI area). (**b**) Schematic of a pomegranate microparticle before and after electrochemical cycling (in the lithiated state). (**c**) Schematics and pictures of free Si nanoparticles (~80 nm), small secondary particles (~1–2 μm) and large secondary particles (~5 μm) of the same Si nanoparticles. (**d**) SEM images of micrometer-sized Si secondary particles prepared via a microemulsion approach [64,68].

Because of their reduced particle size, the use of nanomaterials can cause many new challenges, such as high surface area, low packing density, and high cost. Rational design of nanomaterials must compensate for these associated disadvantages while addressing the problems of materials of micrometer size. During battery cycling, the formation of the SEI layer on the electrode surface consumes the cathode electrolyte and lithium. Compared to electrodes made up of micrometer-sized materials, the SEI forms part of the surface of nanostructured electrodes, due to the much higher electrode–electrolyte interfacial area, consumes more electrolyte and lithium, resulting in low initial Coulombic efficiency and significantly reduced overall battery capacity and energy density. A stable SEI is critical to the electrode’s long cycle life while controlling the specific SEI (electrode/electrolyte surface area) plays an important role in achieving high initial Coulombic efficiency. The problems of SEI’s large volume expansion and instability for high-capacity electrode materials are solved by engineering nanostructures with an electrolyte-blocking layer and an internal void space. However, due to their high surface area, the problem of low initial Coulombic efficiency arises for these nanostructures. The high surface area of electrode/electrolyte increases the risk of severe side reactions involving decomposition of electrolytes and consumption of lithium. High surface area is an inherent feature of nanomaterials, but by engineering their secondary structures, the electrode/electrolyte surface area can be adjusted. Figure 2a shows the calculation of a specific area of SEI for a secondary particle of a micrometer scale consisting of assembled nanoparticles. The specific SEI area can be reduced significantly with an electrolyte-blocking layer outside the assembled secondary particles [64]. Thus, the use of micrometer-scale secondary particles, compared to primary nanoparticles, can help to reduce deferred side reactions between the electrolyte and the electrode and achieve higher initial Coulombic efficiency. Of noteworthy success are Si anode materials with a nanoscale design inspired by grenades with the following characteristics (Figure 2b): micrometer-sized Si secondary particles composed of primary Si nanoparticles; each primary Si nanoparticle has a carbon shell with void space, and each secondary particle has an outer carbon layer as an electrolyte-blocking layer [64]. 

In addition to the reduced specific SEI, the interconnected carbon framework provides fast electron transport pathways. Vacuum space can mitigate Si’s volume expansion on lithiation. These primary particles also allow for high tap density and volumetric mass loading, which are important parameters for electrode evaluations. Decreasing the particle size to the nanometer scale creates a lot of interparticle space, which usually results in low tap density for materials and therefore the low volumetric capacity for an electrode. Because of their significantly high surface energy, nanoparticles tend to bridge/aggregate strongly into secondary microparticles. In addition to the interparticle space between secondary microparticles, there is a large interparticle space within the aggregation of nanoparticles, resulting in a higher overall porosity compared to that of the interparticle space materials between individual microparticles. Meanwhile, the reduced particle size also induces large resistance of interparticle (relative to the same mass loading), which creates barriers in the electrode for electron transport. Thus, using nanostructured materials, achieving electrodes with high mass loading and areal capacity is challenging. Because of the reduced interparticle space, the tap density of micrometer-sized particles is generally higher than that of free nanoparticles. As such, by engineering a micrometer-scale secondary particle/cluster densely assembled by small primary nanoparticles, the tap density of nanomaterials can be significantly improved (Figure 2c) [64]. 

With several advantages, the nanomaterials also possess some disadvantages in the Li-ion battery applications, such as:

(i) This increase in surface area will also increase the undesirable side-reactions and potentially decrease the safety of the cells. For example, when nanomaterials are utilized as the cathode in lithium-ion batteries, their increased surface area increases reactions between electrode and electrolyte, which results in increased irreversibility but to compromise with the poor cycle life. In addition, the high surface reactivity of nanoelectrode materials causes poor cycle life. For example, in olivine LiFePO_4_, Fe^2+^ is quite unstable in the atmosphere where O_2_ and a small amount of H_2_O coexist. The stability of LiFePO_4_ in air atmosphere decreases with the reduction of particle-size since nanosize increases the number of molecules exposed in the air [65]. Okubo et al. demonstrated the good high-rate performance of nanocrystalline LiCoO_2_ up to 100 °C, although extreme size reduction (below 15 nm) proved detrimental to cathode performance [57]. The cycling performance of nanosized LiCoO_2_ was, however, much inferior to that of a bulk sample, which they attributed to the enhanced reactivity of the high surface area material. 

(ii) The volumetric energy density of lithium-ion batteries is quite lower in nanoelectrode materials compared to microsized materials [51]. 

#### 1.5.1. Comparison of Bulk and Nanomaterials for LIBs

The micrometer-sized LiNi_1−x_MxO_2_ secondary particles composed of aligned needle-like nanosized primary particles [66] and LiFePO_4_ secondary particles containing nanoscale carbon-coated primary particles [67] are two examples of the most successful designs for intercalation-type cathode materials. Unlike these cathode insertion materials, however, engineering the void space for each primary particle is crucial to achieving the long cycle life of high-capacity electrode materials due to their large volume lithiation expansion. Based on the principles of secondary cluster design, we have recently shown that high tap density can be achieved for powders made up of micrometer-sized Si secondary particles with a pomegranate—like a nanostructure and Si secondary clusters made using a mechanical approach (Figure 2d) [64,68]. The Si secondary cluster tap density produced by the mechanical approach is 0.91 g cm^−3^, six times the primary nanoparticles density (0.15 g cm^−3^). Important aspects to note here are the integration of carbon nanotubes into the Si clusters, which helps improve the electrical conductivity of the inter-cluster in the electrode, while the interconnected carbon network provides the pathways for fast electron transport within a secondary cluster. High areal mass loading is thus achieved for the as-obtained electrodes due to the good conductivity. As expected, for the as-prepared Si clusters [68], stable cycling with a high areal capacity of ~3.5 mAh cm^−2^ at a high areal mass load (>2 mg cm^−2^) is achieved. Therefore, both high tap density and mass loading were achieved due to space-efficient packing of nanometer-sized primary nanoparticles within the micrometer-scale secondary particles and the interconnected carbon frame within each secondary particle. The current strategy of engineering micrometer-scale secondary particles and an interconnected conductive network is both easy and effective in solving the critical issue of low tap density and nanomaterial mass loading, and it can be extended to prepare various high-capacity electrode materials.

### 1.6. Challenges for the Utilization of Nanomaterials for Li-Ion Batteries

Even though nanostructured anode materials exhibit better electrode performance, there are some limitations for commercial applications. The coulombic efficiency and reversible capacities of nanostructured anode materials are much lower than the bulk samples. For example, in nanosized LiCoO_2_, the coulombic efficiency was decreased to 26% and reversible capacities were decreased to 65 mAh/g from >140 mAh/g [57]. Since the coulombic efficiency and reversible capacities are decisive factors for electrodes in commercial applications, a great focus can be placed on how to obtain more stable surfaces and better crystallinity of nanomaterials for their practical utilization in batteries. Therefore, it is essential to use the nanomaterials into smart nanostructures with tailored properties, which specifically improve the desired reaction. Choi et al. [69] deduced a novel method to create anode and cathode materials with controlled properties. Another important challenge in synthesizing nanomaterials is the production cost. The new technology for large-scale production show cost low cost with lightweight materials. It is important to encourage utilizing renewable energy sources possessing low cost highly efficient batteries. 

## 2. Carbon-Based Nanostructures as Anode Materials for Li-Ion batteries

In a battery, loss of electrons takes place in the anode. The electron extracted from the anode is utilized as electricity through an external electric circuit. In general, a negative electrode in a primary cell is called the anode and the positive electrode is called the cathode. The preferred characteristics of the anode materials are high reversible capacity pleasing charge profile, advantageous kinetics (rate capability), longer cycle life and longer calendar life, effortlessness of processing, safety, compatibility with the electrolyte solution, and cost-effective. In the initial ambient-temperature rechargeable lithium cells, the negative electrode was metallic lithium. The important capacity loss that results from the reaction of metallic lithium with the organic solvent liquid electrolyte and the severe safety problems that results from the periodic deposition and dissolution of metallic lithium during cycling sharply diminished the commercialization of these cells. Replacement of the metallic lithium by lithium-intercalation compounds enhanced both cell cycle life and safety [70]. 

### 2.1. Carbonaceous Materials as Anode Materials for Li-Ion Batteries

The lithium-ion cells that are commercially available utilize carbon-based materials for their negative electrode. A breakthrough occurred in 1983, where the Li metal anode was replaced by a graphite-based carbon material in which lithium was reversibly intercalated and deintercalated [71]. Carbon-based materials are preferred for the anode, because of their availability, low cost, and performance. In addition, carbon materials protect the lithium from dendrite formation while recharging the battery. Carbon materials are available in various forms, such as graphite, hard and soft carbons, carbon fibers, carbon nanotubes, etc. Usually, commercial Li-ion batteries use graphite as the anode material due to its outstanding stability. However, their theoretical maximum capacity is limited to 372 mAh g^−1^, corresponding to the formation of LiC_6_ [72]. Its poor cycling behavior, a consequence of the easy movement of the graphene planes along the a-axis direction during the intercalation and de-intercalation of lithium, in addition to the problem of solvated lithium intercalation, have since ruled this material out of practical lithium-ion battery applications. Other methods for modifying synthetic and natural graphites have been tested, e.g., gas-phase oxidation by oxidizing gases, for example, oxygen, air, and ozone, but it is difficult to obtain satisfactory anode materials on a tonnage scale since the uniformity of the product is not easy to control. The use of nanostructured materials as electrodes in cells, in place of conventional electrodes, was expected to give higher lithiation capacity and better execution due to the nanomaterials’ high surface area compared to bulk materials. Henceforth, the abundant research effort was focused on choosing nanomaterials appropriate for this application. Carbon nanomaterials in various morphologies, such as carbon nanotubes, carbon nanofibers, carbon xerogel, carbon nanosprings, carbon nanorods, etc. were tested as anode materials.

### 2.2. Carbon Nanotubes (CNTs) as Anode Materials for Li-Ion Batteries

Since the discovery of carbon nanotubes in 1991 [73], scientific and technological interest has been stimulated intensively in the field of synthesis and applications of various CNTs and modified CNTs. CNTs are composed of rolled graphene sheets, which resemble building “bricks”. The basic constitution of the nanotube lattice is the strong C–C covalent bond (as in graphite planes). The perfect collection of atoms on the lattice along the tube axis and the closed topology endows nanotubes with in-plane properties of graphite, such as elevated conductivity, excellent mechanical strength, stiffness, chemical specificity, and most of all, its inert nature. Moreover, the nanosized structure gives a wide surface area which helps in enhancing the vast application in the field of mechanical and chemical engineering.

**Figure 3:**
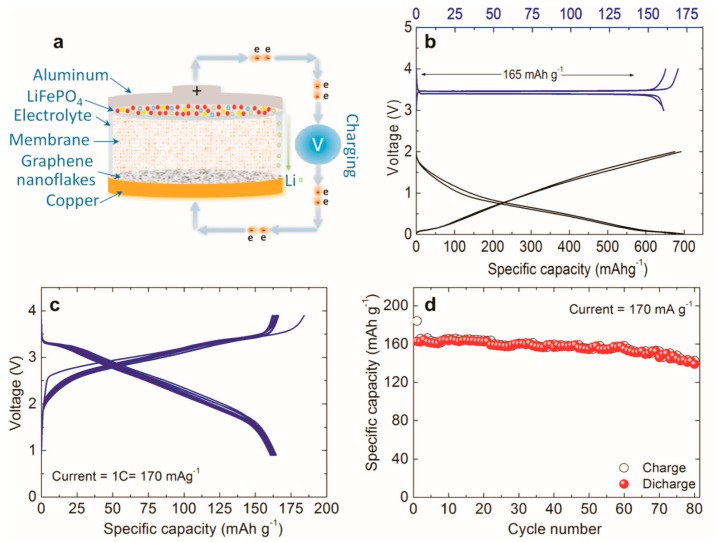
Electrochemical profile of graphene /lithium iron phosphate lithium ion cell. (**a**) Schematic diagram of graphene/lithium iron phosphate cell. (**b**) Charge-discharge voltage profiles of the single electrodes (**c**) Voltage profile of the graphene/LiFePO_4_ full cell. (**d**) Specific capacity versus cycle number of the cell [74].

Carbon nanotubes are classified in several ways based on their configuration, degree of graphitization, and structure. Based on configuration, it can be classified into an armchair, zig-zag, and chiral. Depending on the degree of graphitization, they can be classified into amorphous and graphitic. depending on the structure, they can be classified into single-walled (SWCNT) and multiwalled carbon nanotubes (MWCNT). The electrochemical properties of CNTs (both MWNTs [51,52,53,54,55,56,57,58,59,60,61,62,63,64,65,66,67,68] and SWNTs [51,57,65,66,67,68]) used as the anode materials for lithium-ion batteries were investigated extensively and it was found that lithium intercalation/deintercalation in the hosts was dependent upon the morphology and structure of the CNTs. A single graphene sheet rolled into a cylinder with an almost uniform diameter in the range of 1–2 nm and lengths of several micrometers form SWNTs. Each rope contains many tubes, ranging from 203 to several thousand. The surface of SWCNTs is much higher with a value of 2630 m^2^/g. A lithium-ion battery with a graphene ink anode exhibited stable operation for over 80 charge-discharge cycles of 165 mAh g^−1^ with an energy density of around 190 Wh kg^−1^ (Figure 3) [74].

**Figure 4:**
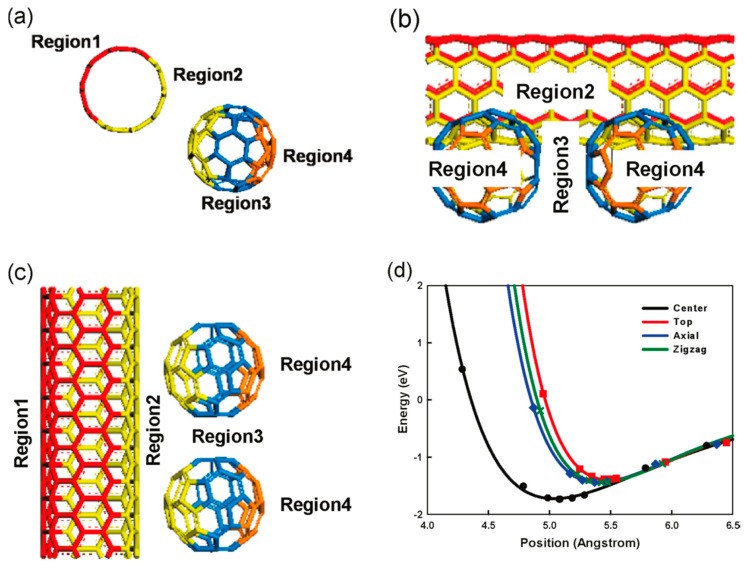
Elementary structure of (5,5) CNT-C60 hybrid system: (**a**) front view; (**b**) side view; (**c**) top view; region 1-red, region 2-yellow, region 3-blue, and region 4-orange; (**d**) Single point energy calculation of the Li atom on the different positions of the hexagonal ring in the (5,5) SWCNT [80].

Graphitic sheets are rolled into closed concentric cylinders to form MWCNTs [75,76,77,78]. These MWCNTs showed higher capacity than graphite. The conduction properties in MWNT are significant, and ballistic electron transport and the Aharnov–Bohm effect were observed in individual MWNTs [79]. The distinctive structural arrangement of MWCNTs resulted in a superior capacity to graphite. The aligned MWCNTs exhibited higher lithium storage (980 mAh/g) than in-aligned MWCNTs (158 mAh/g). After ten cycles, the capacity loss was observed in both cases, but the loss in capacity was much higher in nonaligned MWCNTs compared the that of aligned MWCNTs. The SWNTs showed reversible Li capacity on the order of 460 mAh/g, which is higher than the theoretical value for graphite (372 mAh/g). Li insertion leads to irreversible structural disorder inside the CNT rope lattice. Figure 4 illustrates the Li adsorption mechanism on the CNT-C60 hybrid system [80]. 

This suggests that Li ions intercalate in the channels between nanotubes and disrupt the intertube binding, in contrast to the well-ordered doping superlattices observed in graphite, polyacetylene, and C_60_ hosts. For Li-ion battery applications, SWNT can be a promising material due to its high reversible capacity and high rate performance. The limiting factors of these nanotubes in battery application so far are the large irreversible capacity and the large voltage hysteresis. The electrochemical performance of CNTs depends on the presence of impurities, structural defects, and nature of graphitization, and these are controlled by their preparation methods and the treatment process. The further treatment process includes purification, ball milling, an etching process, acid treatment, etc. and can be adapted to enhance the electrochemical performance of the CNTs [81,82]. 

**Figure 5:**
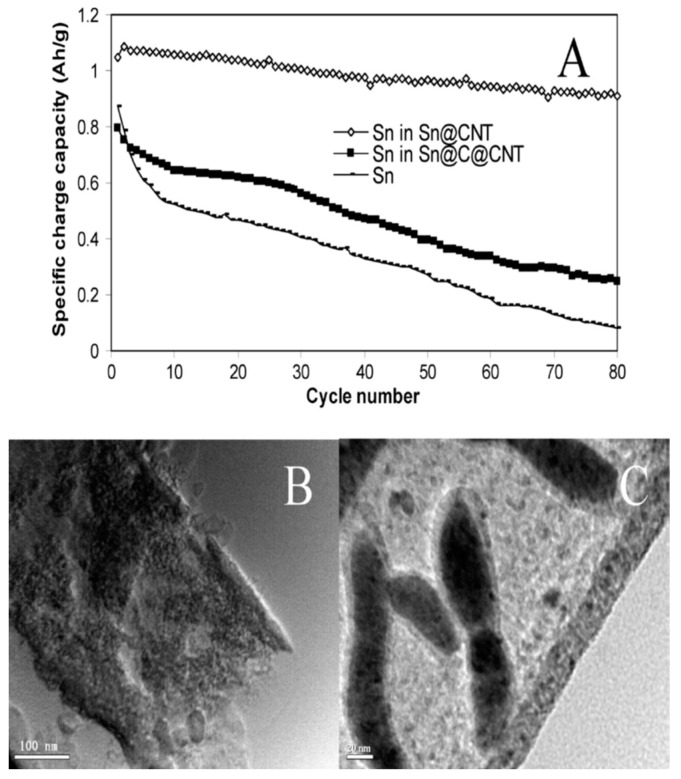
Electrochemical properties of Sn contributions in Sn@CNT and Sn@C@CNT (**a**); TEM image of Sn@CNT electrode after 80 cycles (**b**); TEM image of Sn@C@CNT electrode after 80 cycles (**c**) [86].

The electrochemical performance of CNT could be improved by modifying the CNT structure by adding additives. The porous carbon nanostructure α-CNT showed a reversible capacity of ∼302 mAh g^−1^ and exhibit little hysteresis in the charge-discharge process [83]. CNT with quadrangular structure and one open end have higher reversible capacity compared to the common multiwalled carbon nanotubes [84]. The initial capacity of carbon nanotubes can be improved by constructing carbon nanotube networks onto carbon fiber (CNT/CFP) [83,84]. The improved performance is due to the electroactive area introduced by carbon fiber on CNT. To minimize the electrode weight and size, a simple paper electrode was prepared by using “free-standing” single wall carbon nanotubes (SWNT) [85]. These paper electrodes exhibited a slightly lower capacity compared to conventional electrodes. However, the capacity of this paper electrode could be improved by using carbon black additives. Comparison of electrochemical performance of the CNT in single-wall (SWCNT), double-wall (DWCNT), and multiwalled (MWCNTs) structures demonstrate that both SWCNT and DWCNT films exhibit more specific charge of 2390 mAh g^−1^ and 2110 mAh g^−1^ compared to the MWCNT film with a value of 750 mAh g^−1^ (Figure 5a). However, strong capacity fading was observed with SWCNT and DWCNT films. the MWCNT film has higher coulombic efficiency than both the SWCNT and DWCNT films. The active phase in CNT improves the electrochemical properties. By adding tin with CNT enhanced the charge capacity of CNY anode (Figure 5a). Figure 5b,c presents the TEM image of Sn@CNT electrode and Sn@C@CNT after 80 cycles, respectively [86].

Maurin et al. [87] examined the MWCNT gotten by an electric-arc method. The sample contains multiwalled carbon nanotubes MWNTs, onions, and amorphous carbon. The littlest nanotubes have two concentric graphitic shells. Diameters differ from 5 to 20 nm with a mean estimation of 15 nm, and lengths are >1 μm. The ends of the nanotubes are constantly capped. These carbon nanotubes display a reversible capacity of 180 Ah/kg, contrasted with 372 Ah/kg in graphite. The reversibility concerns the amount of intercalated lithium that can be deintercalated and not the thermodynamically or kinetic parts of the intercalation procedure. This capacity is retained after ten cycles and the proportion discharge capacity–charge capacity is constant and is equal to 1.02 ± 0.01. The irreversible capacity is of 145 Ah/kg. The low electrochemical specific capacity estimated is probably a result of both the confinement of the Li species that makes a ‘bottleneck’ which hinders the lithium dispersion and the low extent of MWNTs in the raw material. In the spectral changes of the Raman spectrum, the Raman E_2g_ band defines the interlocked Li-ion between the graphene layers of the carbon nanotube without the progress of n-staged phases with n higher than two and further proceeds by interlocking graphite compounds [88].

Gao et al. [89] compared single-walled carbon nanotube (SWNT) electrochemical performance for samples equipped by laser ablation technique and by further purification methods. It was observed that the SWNT exhibited a reversible saturation composition (RSC) of 450 mAh g^−1^ for Li_1.2_C_6_ by laser ablation technique. By further removing the impurity phases by filtration, the RSC raised to 600 mAh g^−1^ for Li_1.6_C_6_ which was apparently larger than the ideal value of 372 mAh g^−1^ for LiC_6_ in graphite. In the case of MWCNTs prepared by catalytic decomposition, the as-prepared MWCNTs without purification show high reversible capacity. After purification with a solution of hydrofluoric acid and then refluxing with nitric acid and heat treatment, irreversible capacity decreased. This change in the irreversible capacity was also associated with a decrease of the reversible capacity, from 390 mA h/g for purified as-prepared MWNTs to 230 mA h/g for the MWCNTs heat-treated at 2800 °C. By comparison to graphite, such values correspond to Li_1.04_C_6_ and Li_0.62_C_6_, respectively [90]. 

The raw carbon nanotubes were oxidized at a temperature of 130 °C, using a mixture of nitric and sulfuric acid to improve its storage capacity. A total discharge capacity of approximately 660 mA hg^−1^ was obtained after the initial discharge and in contrast, the storage capacity wads 200 mA hg^−1^. This abrupt increase in the storage and leaching capacities were because of the effect of acid-oxidation on carbon nanotubes. This increased storage capacity is because of the intercalation procedure. Initially. The residuals in the acids caused them to react with the Li ions and cause the irreversible capacity. The nitric and sulfuric acid can easily infuse through the layers of the graphene sheets, creating defects such as pores in the graphene sheets and ultimately help in the expansion of graphite. Therefore, Li atoms can now easily diffuse into the graphite layers through defect sites produced by oxidation, hence increasing the reversible capacity [91]. 

The prepared nanotubes are subjected to the ball-milling process. The electrochemical properties and hence the battery performance was changed after ball-milling. The reversible capacity of lithium ion batteries was enhanced to 1000 mAh g^−1^ with Li_2.7_C_6_; at the same time, the irreversible capacity of lithium was diminished from Li_3.2_C_6_ to Li_1.3_C_6_ [92]. Efforts were taken up by various researchers to understand the causes for lower irreversible capacity in ball-milling nanotube and illustrate few models to enhance the electrochemical properties. Xing et al. [93] reported that in ball-milled sugar carbons, the reversible capacity of lithium is increased from Li_1.5_C_6_ to Li_2_C_6_. The lithium insertion in ball-milled sugar carbons is enhanced by carbon radicals present at the edges of graphene sheets with fractured structure. Disma et al. [94] studied that using Li_1.9_C_6_ that extended ball-milling time (80 h) to the sample produces a defect center along the graphite c-axis and breaks the graphene layers. The attributed large reversible capacity was designed due to the adsorption of Li ions on two sides of the separated graphene layers. 

The development of the microtexture and the composition of the nanotubes with the thermal treatment, from 900 to 2800 °C, permitted the investigation of the connection between the physical and the electrochemical behaviors of MWNT. The microtextural irregularities in the tube walls cause the catching of lithium at the edges of pseudographitic aromatic layers of MWNT and are completely responsible for the irreversible sorption phenomenon. Heat treatment of the nanotubes provided the excellent textural organization with a reduction of the surface area and gradual removal of the heteroatoms, leading to limited lithium inclusion, which is reversible as well as irreversible. The difference between the decrease and the oxidation overvoltage somewhat reduces for MWNT treated at 1600 °C and for MWNT 2000 °C. Above 2500 °C, that is, after almost full graphitization, the electrochemical storage in carbon nanotubes continues nearly without hysteresis; however, the value of the capacity is very low (<100 mAh g^−1^). It was expected that the central cores of MWNT play an important role in the cumulation of ions as well as for the creation of an electrical double layer. Henceforth, their closing with thermal treatment and the formation of continuous carbon layers is unfavorable for lithium sorption [95]. 

The CNT structure could also be modified by a chemical etching process which could at once change the electrochemical performance of CNT’s. The shorter single-walled carbon nanotubes produced by chemical etching improve the Li diffusion into the interior of CNTs, thereby enhancing the reversible capacity of CNTs. Nevertheless, the coulombic efficiency was reduced by large structure defects [96]. The hysteresis could be reduced by using shorter single-walled carbon nanotubes [97]. Yang et al. [98] compared the electrochemical performance of long and short CNTs. The reversible capacities of short CNTs were twice those of long carbon nanotubes. Also, the charge-transfer resistance of short CNTs was much lower than those of long carbon nanotubes. As a result, the short CNTs revealed higher electrochemical performance during the charge and discharge process. With the aim of miniaturizing electrode devices, ultrashort CNTs were prepared by Wang et al. [99]. These micrometer-length CNTs showed much better electrochemical performance in Li-ion batteries. Introducing many defects and pores by etching increased CNT capacity [100,101,102,103]. The structural analysis of closed and opened carbon nanotubes showed that the closed carbon nanotubes possess pentagons and heptagons with capped tube end in which insertion of Li-ion is not possible into the inner core of the tubes. On the other hand, the Pentagon and heptagon structures were absent in open tubes and so Li-ion insertion into the inner core of the tubes is possible and this lithium cannot deintercalated. Therefore, the opened carbon nanotubes showed poorer cycling performance than the closed carbon nanotubes [104,105].

The degree of graphitization shows a significant role in the electrochemical behavior of MWCNTs. The charge capacity of the somewhat graphitized carbon nanotube is higher (640 mAh/g) than that of the well-graphitized carbon nanotube (282 mAh g^−1^ during the first cycle). The higher charge capacity of the slightly graphitized carbon nanotube sample could be related to Li being doped mostly into regions without organized graphitic structures, microcavities, edges of graphitic layers, and surfaces of single graphitic layers. Together, they showed a voltage hysteresis of about 1 V, which is higher than for hydrogen-containing carbon. However, the graphitized MWNTs showed better cycle life and rate capability. The stable structure is because of the reduction in charge capacity 65.3% their initial value at 20 charges–discharge cycles. The well-graphitized samples sustained 91.5% of their original charge [106]. The CuO/MWCNTs, on the other hand, could supply 700 mAh/g [107]. Less graphitized MWCNT was compared with more graphitized MWCNT structure. It was inferred that the properties of specific capacity and cycle stability were enhanced in less graphitized MWCNT. Cycle stability and charge-discharge rates were enhanced in the more graphitized MWCNT structure [108].

The electrochemical performance of CNTs is improved by doping with various heteroatoms. For instance, boron-doped multiwalled carbon nanotubes (B-MWNTs) exhibit higher reversible capacity than undoped multiwalled carbon nanotubes [109]. The resistivity of bulk samples decreases by alkali doping [110]. The internal and external surfaces of the SWNT ropes were decorated by alkalis [111]. Tangential mode shifts result from electron transfer from alkali dopants to the SWNT [112]. Cobalt oxide nanoparticle coated on CNT surface exhibited good electrochemical performance through a conversion reaction [113]. 

All the electrochemical reactions occurring in a CNTs–Co_3_O_4_ core-shell nanocomposite anode can be described as follows (Equations (8) and (9)):

8Li^+^ + Co_3_O_4_ ↔ 4Li_2_O + 3Co (conversion reaction) (8)

xLi^+^ + C (CNT) ↔ Li_x_C (intercalation reaction) (9)

Another example is carbon nanotubes coated with a layer of copper oxide (CuO/CNT) by a plating method followed by oxidation at 160 °C in air. They showed a reversible capacity of 700 mAh g^−1^, corresponding to 1.88Li for 6C, but the high capacity exhibits a larger potential hysteresis. The Li insertion properties of CuO/carbon nanotubes showed that CuO in CuO/carbon nanotubes can reversibly store 268 mA h Li g^−1^ CuO. Here, Li inserts into the CuO lattice at 1.7 to ~1.0 V and is released at 2.3 to ~2.5 V vs. Li, according to the following equation (Equation (10))

CuO + xe + xLi ↔ CuOLi.(10)

The cycle life of Li/CuO/CNT cell is limited due to CuOLix decomposition to Cu, Cu_2_O, and Li_2_O and the microstructure of CNT was destroyed during Li insertion and release [114]. Yang et al. [115] confirmed the diffusion of Cu^2+^ ions into the pores and onto the exterior surface of the carbon nanotubes. The transformation of Cu^2+^ ions to Cu takes place at elevated temperatures in the carbon nanomaterial. Hence, the pores present the CNT and the exterior surfaces will be filled partially with Cu atoms until all the pores are occupied. Consequently, the carbon nanotubes doped with Cu will possess a lower specific capacity.

Metals such as Al, Sn, Sb, Pb can store Li by means of combination development through electrochemical means, bringing about higher capacities than those of carbonaceous materials. A huge specific volume change likewise happens amid Li addition and extraction which cause the electrode to flop by pummeling. Accordingly, quick capacity blurring is observed [116]. Thus, to improve the structural stability, carbon nanotubes (CNTs) doped with Sb and SnSb particles were prepared by chemical methods using SnCl_2_ and SbCl_3_ as precursors along with CNTs. CNTs along with Sb and SnSb particles exhibited enhanced cyclability and reversible specific capacities in contrast with pure CNT’s. The reversible capacities for CNT-36 wt.% Sb were found to be 462 mAh/g and for CNT-56 wt.% SnSb the reversible capacities were as high as 518 mAh/g.

The enrichment in cyclability is connected to the nanoscale features of the metal nanoparticles and CNTs possess a unique role involved in the mechanical stress induced by specific volume changes in electrochemical Li addition and extraction reactions [117]. Sn/CNTs [118], Si/CNT [119], SnO_2_/CNTs [120], and SnO^2^/CNTs [121] composites possess larger capacity and enhanced cyclability than pure metals or metal oxides. Shu et al. [122] fabricated a cage-like CNTs/Si composite structure, exhibiting a large reversible capacity of 940 mAh g^−1^ and enhanced cycle performance. The Sn-encapsulated CNT nanostructure (Sn@CNT) exhibited high reversible capacity and good cyclability [86]. Both Sn@CNT and Sn@C@CNT anodes, even after 80 cycles of discharging and charging, retained their tubular structure and no significant growth in particle size was observed during the cycling period (Figure 5) [86].

It is predictable that in metal (oxide)–CNT composites, the volume variation that occurs in both metals or metal oxides can be more efficiently lodged in CNT during the process of charging and discharging. The encapsulation quantity of SnO_2_ in CNTs is directly related to reversible capacity and cycle performance of SnO_2_/CNT composite. Appropriate entrapping of SnO_2_ exhibits a reversible capacity as high as 383 mAh g^−1^ with subsequent cyclability with only 0.4% capacity loss/cycle. Appropriate entrapping of SnO_2_/CNTs could not only provide high electrical conductivity but also successfully accommodate the volume variations of SnO_2_ during the cycling processes [121].

For MnO_2_, a conventionally used cathode material, very few reports are available on its use as an anode material for Li-ion battery cells [123]; however, it has been reported to attain large reversible capacity as an anode material [124]. Furthermore, MnO_2_–CNT composite structures have been proved to be an efficient way to progress electron transfer for use in supercapacitors [125]. As stated by Reddy et al. [126] even though the MnO_2_/CNT composite electrodes can distribute a reversible capacity of about 500 mAh g^−1^ after 15 cycles, a faster decay capacity within 15 cycles was observed. 

The Caterpillar-like nano flaky exhibits an Even larger reversible capacity of 801 mAhg^−1^ was observed for MnO_2_/carbon nanotube (CNT) nanocomposites [127], with ~1000 mAhg^−1^ contribution due to the MnO_2_ porous layer alone for the first cycle with a reasonable rate capability. The electrochemical performance when compared to the pure MnO_2_ electrode, unique hierarchy architecture in MnO_2_/carbon nanotube (CNT) enhanced the property, thereby providing faster lithium ion and electron transport and to fulfill the large volume change during the conversion processes.

### 2.3. Carbon Nanofibers as Anode Materials for Li-Ion Batteries

**Figure 6:**
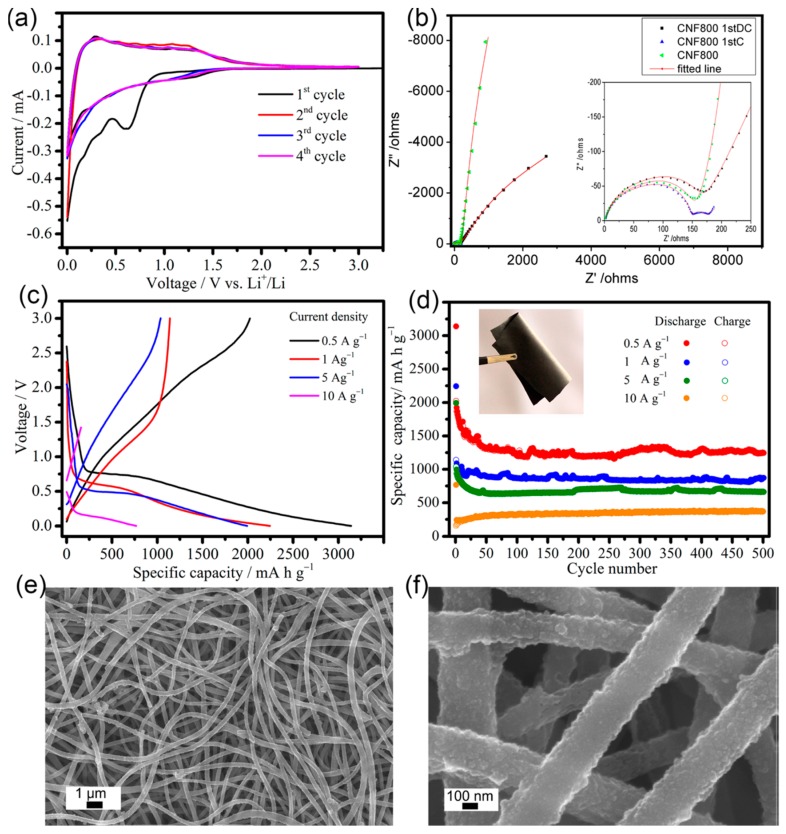
Cyclic voltammetry (CV) profiles (**a**) scan rate of 0.1 mV s^−1^ and EIS profiles (**b**) CNF800- anode material in pure state (CNF800), the first discharging (CNF800 1stDC) and first charging state (1stC). The initial charge/discharge curves (**c**) and the cyclic performance (**d**) of CNF800 at many current densities (the inset is a digital photo of CNF800 film). The SEM images of CNF800 as anode film at the 500th discharge state (**e**,**f**) [129].

An encouraging anodic material is carbon nanofiber due to its ability to highly graphitize at low temperatures and its reduced cost of bulk production. The rate of graphitization and their structure are greatly important for the anodic performances in Li-ion batteries. Yoon et al. [128] produced highly graphitized carbon nanofibers (CNFs) by CVD at 550–700 °C and could control the structure from platelet (P) to tubular (T) at such elevated temperatures. Such CNFs exhibit high capacity of about 297–431 mAh/g, particularly in the low potential areas. The coulombic efficiency in the first cycle is nearly 60%. Doping nitrogen is another approach to enhance the electrochemical properties of carbon materials. Wang et al. [129] observed that flexible CNF films with nitrogen or oxygen dopants had deformities in graphite structure and can display initial capacities of 2000 and 755 mAh g^−1^ corresponding to current densities of 5 and 10 Ag^−1^, respectively. Even after 500 cycles, CNF800 retained capacities of about 1251, 865, 702, and 305 mAh g^−1^ at 0.5, 1, 5, and 10 Ag^−1^, respectively (Figure 6). 

### 2.4. Carbon Xerogel as Anode Materials for Li-Ion Batteries

**Figure 7:**
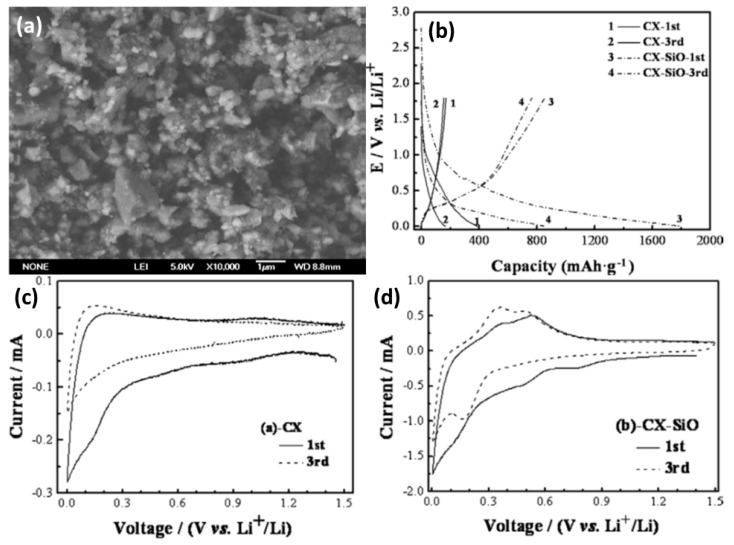
FESEM image of CX–SiO; (**b**) charge-discharge profiles, and (**c**,**d**) Cyclic voltammograms of CX and CX–SiO measured in the voltage range of 0–1.5 V with a scan rate of 0.1 mV s^−1^ [130].

Carbon xerogel (CX) is a unique carbon based-material with a continuous nanoporous structure with low-density. The electrochemical measurements of CX and CX–SiO were correlated by Yuan et al. [130]. CX–SiO consists of active C, graphite, SiO, and dispersed Si crystals, while the CX is made up of only active C and graphite. CX–SiO consists of uniformly dispersed particles in comparison with CX xerogel. The SiO in CX–SiO xerogel can significantly raise the discharge capacity of the CX xerogel. The charge-discharge capacity of CX–SiO comes primarily from the Li insertion–extraction in Si–SiO in the sample. Figure 7 exhibits the typical FESEM image, charge-discharge profiles, and cyclic voltammograms of CX and CX–SiO measured in the voltage range of 0–1.5 V. Studies have confirmed that the prepared CX–SiO composites present themselves as promising anode materials for high capacity rechargeable Li-ion batteries [130].

### 2.5. Carbon Nanosprings as Anode Materials for Li-Ion Batteries

**Figure 8:**
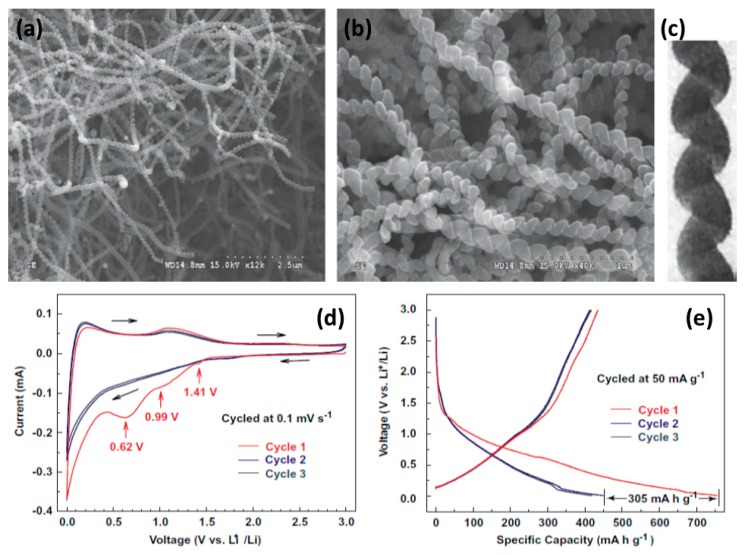
Typical (**a**,**b**) SEM and (**c**) TEM images of carbon nanosprings; (**d**,**e**) Charge-discharge curves of the fabricated device [131].

A novel carbon nanospring (CNS) benefiting from the nanometer-sized carbon ring radius (ca. 50 nm) was synthesized by Wu et al. [131]. The unusual spring-like morphology of CNSs with pitch distances of about 150 nm is a self-accommodating structure with excellent elasticity, which can effectively accommodate the strain of volume variation during Li insertion–extraction and, therefore, lead to excellent cycling performance. The CNSs show extraordinary potential as superior anode materials for rechargeable LIBs with both high rate capacity and long cycle life. At a present density as high as 3 Ag^−1^, CNSs can give a reversible capacity of 160 mAh g^−1^, which is around six times bigger than that of graphite and three times bigger than that of multiwalled carbon nanotubes under a similar current density. After several cycles, there is no substantial capacity loss for CNSs at both low and high current densities. The enhanced electrochemical properties could be associated with the nanometer-sized building blocks and the unusual spring-like morphology (Figure 8).

In another approach, Shao et al. demonstrated the synthesis of a hematite@carbon nanospring (α-Fe_2_O_3_@CNSs) nanocomposite through precipitation followed by a heat treatment process [132]. The synthesized nanocomposites were examined by several techniques to investigate their various properties and were finally utilized as an electrode material to fabricate Li-ion batteries. The authors observed that with increasing the amount of α-Fe_2_O_3_ in the nanocomposite, the specific capacity of the fabricated device was increased, although the cyclic stability and rate capability of the device were degraded. The authors concluded that due to the strong network of CNSs and dispersed α-Fe_2_O_3_ particles, the cycling performance, specific capacity, and rate capability of the fabricated device based on α-Fe_2_O_3_@CNSs were superior [132]. 

### 2.6. Graphene-Based Nanocomposite Anodes for Li-Ion Batteries

For the application of power source in the case of conveyable electronic device, high-capacity lithium ion batteries (LIBs) are being used. In the case of graphite, storage capacity as low as 372 mAh g^−1^ and reduced performance prevents them from being used as marketable anodes. Hence, researchers have continuously highlighted the preparation of innovative carbon-based anodic materials with advanced performance and storage capacity. Quite a few innovative carbon-based anodic materials, such as carbon-based nanotubes and graphene-based composite materials, have been introduced with high storage capacity, even beyond 1000 mAh g^−1^, with high performance [133,134,135,136,137,138]. Nevertheless, owing to their intrinsically larger surface area and despite suffering from lowered coulombic efficacy, they can attain a capacity exceeding 2 V, which is not satisfactory for use in LIBs. The reason behind the elevated storage capacity in these carbon-based materials is not clearly understood, even though it has been proposed to be due to larger interlayer design, reduced diameter, and increased surface area, etc. [139,140]. Because the density of carbon-based materials is low they have poor storage capacity. 

Incorporation of dopants is one method for controlling the physical and chemical characteristics of a material. With the swift evolution of elastic and wearable electronic devices, the need for stretchable anodes is increasing. Aerogels made of graphene [141,142,143], carbon nanotube foam/paper [144,145], and fiber cloth made of carbon [146,147] were used as self-supported LIB electrodes. These flexible anodes still suffer from low flexibility. Therefore, this remains a challenge. 

Establishing modern techniques for obtaining renewable energy by synthetic paths has been recognized as a new research area [148,149,150,151,152,153]. The proposal of transition metal oxides for energy storage [154,155,156] has been given a lot of attention. Superimposing transition metal oxides on carbonaceous materials, such as reduced graphene oxide (rGO), opens challenges of creating an efficient electrode possessing optimal material characteristics [157,158]. Superimposing CeO_2_ with carbonaceous material results in uniquely enhanced characteristics. Thus, the predicted new hybrid materials possess both the characteristics of the carbonaceous material (high power density and long cycle life) and the transition metal (large energy density) [159,160]. Doping of materials is therefore projected to yield hybrid electrode materials with enhanced power density, energy density, stable cycling, and cycle life.

**Figure 9:**
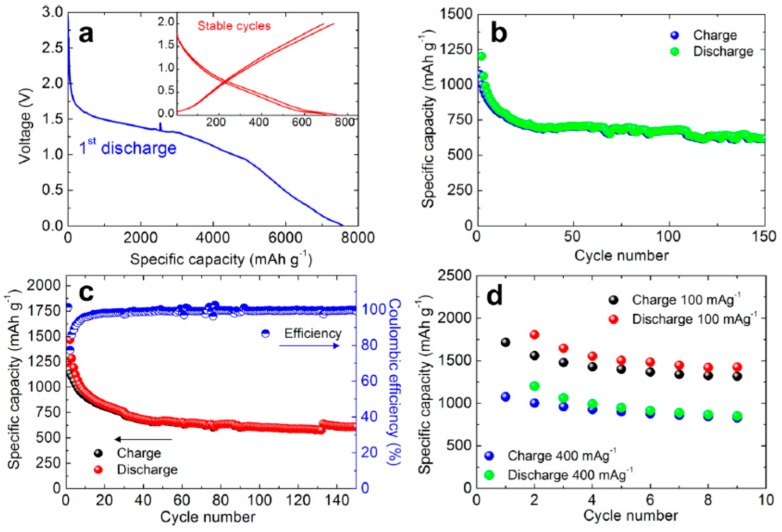
The electrochemical properties of a Cu-backed graphene electrode of a lithium cell. (**a**) Profile of initial discharge (inset: 50th cycle reversible steady-state profile) and (**b**) cycling response of the Li cell for; 700 mAg^−1^, 0.01−2V voltage limit. (**c**) Coulombic productivity (blue dots) at 700 mAg^−1^, voltage limits 0.01−2 against long cycling (red dots) after ex situ lithiation (**d**) Specific capacity versus cycle number at rates of 100 and 400 mAg^−1^ (Hassoun, et al. 2014 [74]).

Graphene nanoflakes are a good resource for advanced Li-ion batteries. A battery was tested in a semi cell using a graphene electrode doped with Cu. The initial discharge voltage profile achieved a current rate of 700 mA g^−1^ (Figure 9a) [74]. The lithium ions on the graphene flakes form a passive film or solid electrolyte interphase (SEI) on the surface of the carbon [161]. Irreversible side reactions occur in lithium-based cells and are related to the electrode [161]. Figure 9b shows the resulting load–discharge cycle with fading initial capacity which then evens out at approximately 750 mAh g^−1^ [74].

For practical applications, a large initial irreversible capacity is undesirable, and this issue is addressed by an ex situ lithiation process carried out by direct contact with the Cu-supported graphene nanoflake electrode with a lithium metal foil, which is wet by the electrolyte. Initial irreversible capacity is of supreme importance to ensure the symmetry between the anode and cathode. Figure 9c illustrates the cycling report for a ~700 mA g^−1^ current discharge rate for the preheated electrode material. The irreversible capacity virtually disappears for the first cycle of Coulombic productivity, while a small decline is observed over a few subsequent cycles (almost ten), most likely connected with the residual SEI film creation process. In fact, at 150 load–discharge cycles, a steady revocable specific capacity of ∼650 mAh g^−1^ was detected. Figure 9d confirms that the Cu-supported graphene nanoflake electrode has a respectable rate capacity; i.e., 100 and 400 mA g^−1^, respectively. Figure 9d likewise illustrates that the Cu-decorated graphene nanoflake electrode has good rate capacity, as tested with two load–discharge rates. When cycled at a lowered rate (100 mAg^−1^), the electrode has the capacity to dispense a very large alterable capacity exceeding 1500 mAh g^−1^, double the theoretical capacity of graphene [162]. These values are greater than those obtained with other C-based nanostructures, such as GNRs [163], delivering a reversible capacity of ~8825 mAh g^−1^ [163] with a density of 100 mAg^−1^. 

**Figure 10:**
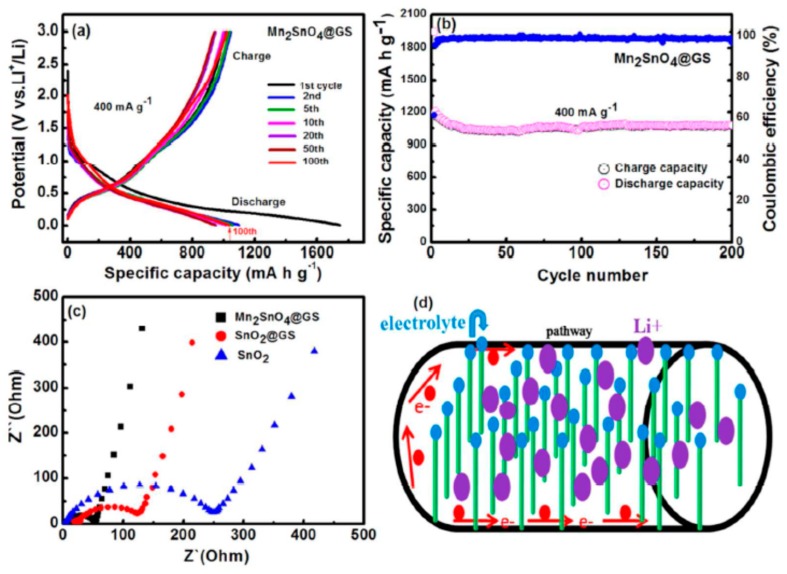
Li loading assets of Mn_2_SnO_4_@GS composite; (**a**) first 100 cycles of expulsion profiles; (**b**) cyclic behavior of Mn_2_SnO_4_@GS at current density 400 mAg^−1^(**c**) Nyquist tin oxide EIS plots, SnO_2_@GS and Mn_2_SnO_4_@GS, (**d**) representative diagram of Li^+^ interaction with e^−^ (Rehman et al. 2018 [164]).

Graphene in the framework performs an influential role in enhancing the anode’s conductivity in diverse phases of the charge-discharge procedure. It also helps to generate a steady (SEI) plate shape that diminishes electrolyte decomposition and facilitates stable cycling performance. Figure 10a reveals the charge-discharge performance of Mn_2_SnO_4_@GS for the initial 100 cycles. After the initial discharge, structural fluctuations occur which gradually become stable after a few cycles [164]. Figure 10b shows the performance of long-term cycling and discharge at a current rate of 400 mAg^−1^. Extraordinarily, Mn_2_SnO_4_@GS showed a reversible capacity of 1069 mAh g^−1^ and 1041 mAh g^−1^ at a current density of 501 mAg^−1^ at 200 and 500 cycles, correspondingly, which is larger than for SnO_2_ and SnO_2_@GS. The electrochemical impedance spectroscopy (EIS) results supplies supplementary data regarding the conversion of charges during the electrochemical reaction intended for the anode in LIBs. The ideal Nyquist plot of SnO_2_, SnO_2_@GS, and Mn_2_SnO_4_@GS electrodes are presented in Figure 10c. Mn_2_SnO_4_@GS displayed a reduced semicircle at high frequency, corresponding to Rct (~60 Ω) compared to SnO_2_@GS (Rct ~120 Ω) and SnO_2_ (Rct ~250 Ω). This indicates upgraded dynamic transference for virtuous electrical interaction and electrode responses in the Mn_2_SnO_4_@GS anode. The sloped curve (Warburg impedance) is referred to as the ion diffusion which occurs at the lower frequency zone. The lower capacity could be responsible for greater Rct of SnO_2_ and SnO_2_@GS. In Figure 10d, the working process of the battery through the charging and discharging of the ternary anode Mn_2_SnO_4_@GS might be due to the collaborative outcome. The Mn_2_SnO_4_@GS and the graphene substrate deliver a larger anode and electrolyte assemblage that constrains particle accumulation, signifying a larger tunable capacity. These ternary terminal substantials are anticipated to limit the distribution of Li^+^ ions and the electrons (e^−^) between the Mn_2_SnO_4_@GS and the collector current grounded on high electronic conductivity graphene sheets. The bouquet-like structure (Mn_2_SnO_4_@GS) helps the electrolytes to be easily admitted and very quickly diffused, enabling superior and stable performance throughout the process [164].

**Figure 11:**
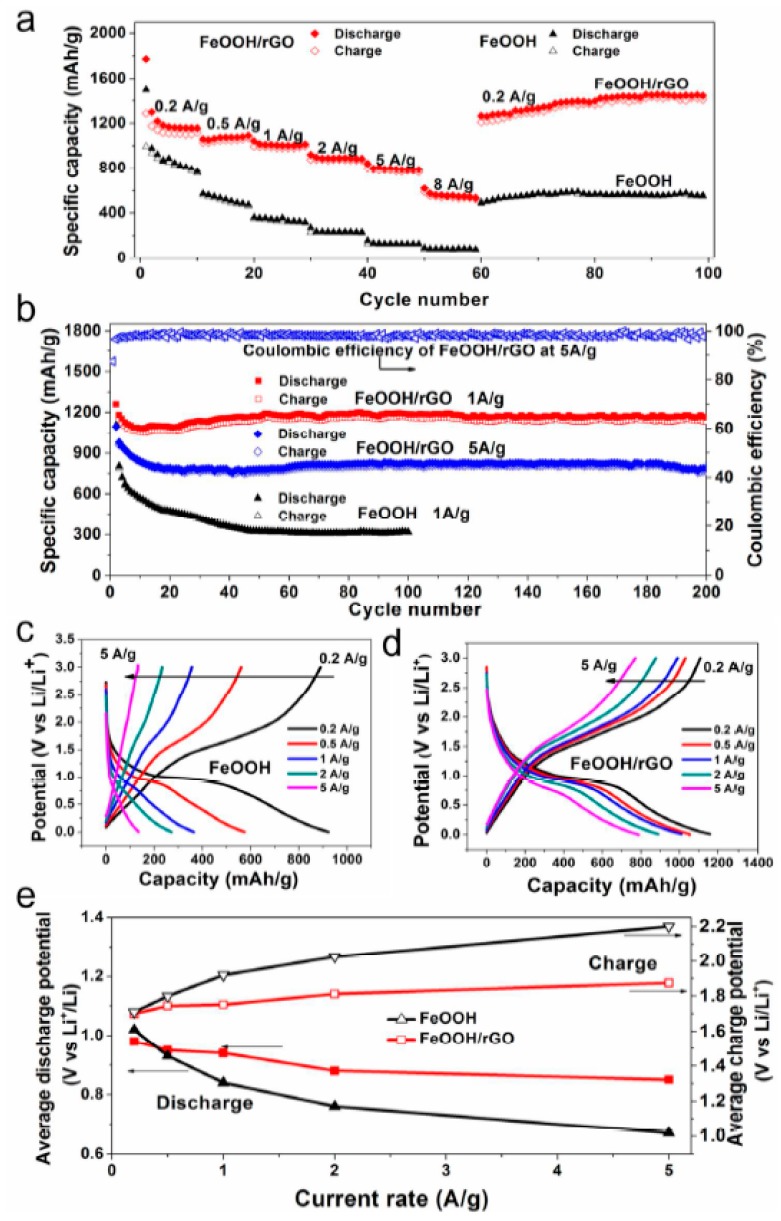
(**a**) Charge rates of unembellished FeOOH and FeOOH/rGO composites for diverse rates (0.2 A/g to 8 A/g), (**b**) Cycling behavior of unembellished FeOOH and FeOOH/rGO composites (first cycled at 0.2 A/g in first cycle), load–discharge curves of (**c**) bare FeOOH and (**d**) FeOOH/rGO composites at diverse rates, (**e**) Typical release and load potential of bare FeOOH and FeOOH/rGO composites at diverse rates. (Hui et al. 2016 [167]).

One category of Li-ion capacitive storage deals with rapid Faradic external redox reaction to propose larger power for electrochemical submissions. Nevertheless, it is quite often restricted due to the condensed amount of energy involvement during the process of charge-discharge, thereby reducing the overall capacity of Li-ion loading in the electrodes [165,166]. Figure 11a shows the rate routine of FeOOH/rGO composites and bare FeOOH with several current densities lying between 0.2 and 8 A/g [167]. FeOOH/rGO composites display larger and steady measurements at diverse current densities with lesser capacity (1156, 1093, 1015, 872, 786, and 535 mAh/g) for an increased current density (0.2, 0.5, 1, 2, 5, and 8 A/g) respectively [167]. It should be noted that the FeOOH/rGO electrode upholds 61.1 percent of the initial reversible capacity at 0.2 A/g, even at a high current density of 5 A/g. In FeOOH/rGO composites from 1263 to 1443 mAh/g, increasing reversible capacity is tested when the current rate returned to 0.2 A/g for 100 cycles. This performance at the initial reversible capacity reaches 112.2% retention of the primary reversible capacity. The observed capacitance of 547 mAh/g and 546 mAh/g was not appreciable and was weakened for current densities of 0.2 and 5 A/g, in the case of the bare FeOOH electrode. The capacitance was increased in a FeOOH-based system when rGO was added to the bare FeOOH electrode. Thus, the composite FeOOH maintained a firmer capacitance throughout the load–leaching process and upheld a faster load transfer rate with colossal energy storage under high current densities (Figure 11b). The outcome shows that after 200 cycles, FeOOH/rGO maintained discharge of 1135 mAh/g—97% of the primary capacity. This performance is obviously superior to that of the bare FeOOH electrode (317 mAh/g after 100 cycles, retaining 40.2% of the initial reversible capacity). Encouraged by this outstanding performance, a larger current density (5 A/g) of the FeOOH/rGO electrode was additionally tested. It was still able to deliver nearly 83.9% of the reversible capacity of 783 mAh/g. The coulombic effectiveness was maintained at 98.4% even after 200 cycles, although the FeOOH/rGO composite showed reduced cycling capacity beyond 20 cycles. These outcomes confirm the good cycling performance of the FeOOH/rGO electrode during the process of ultrafast load–discharge. Figure 11c,d illustrate the FeOOH and FeOOH/rGO discharge profiles for various load–discharge rates. An inclined region (1.0~0 V) and a plateau region (~1.0 V) are detected in these contours, corresponding to the Li-ion surface interface reaction and the Fe(0) conversion reaction, respectively [165]. These contours were compared and examined for various reaction times with the composite material, such as FeOOH and FeOOH/rGO electrodes, and correlated with their capacity performance. The contour for the FeOOH and FeOOH/rGO composite was found in the sloping and plateau region; the performance forcefully ceases for 0.2 to 5 A/g charge-discharge rate as illustrated in Figure 11. Further, the decay indicates that the bare FeOOH electrode fails to maintain both the interfacial and the Li-ion conversion reaction. In addition, mostly the sloping zones in the FeOOH/rGO electrode (Figure 11d) are almost the same for various load–discharge rates with equivalent capacities exceeding 400 mAh/g, signifying their almost unchanged Li-ion storage performance at different reaction rates [166]. Further, the above profiles in Figure 11c,d estimate the gap amongst the charging and discharge process for an individual electrode in the plateau voltages. Thus, changing the gap for various current densities gives a suggestion of the range of polarization in FeOOH and FeOOH/rGO electrodes. As shown in Figure 11e, the FeOOH electrode material apparently expands the potential with the increasing the current rate from 0.2 to 5 A/g. The composite FeOOH/rGO electrode material presents an almost unaffected potential, representing an enhanced electrochemical process at different current densities without noticeable polarization in this electrode. Thus, in general, the overall results demonstrate that the FeOOH/rGO electrode conceivably will not able to deliver enhanced interfacial Li-ion storage on the exterior of FeOOH rods, but at the same time, maintains an ultrafast load transfer rate to promote their performance for various current densities. Furthermore, this performance is cost-effective compared to other FeOOH-based electrode materials. Thus, FeOOH/rGO electrodes show great potential as anodes in LIBs [167].

**Figure 12:**
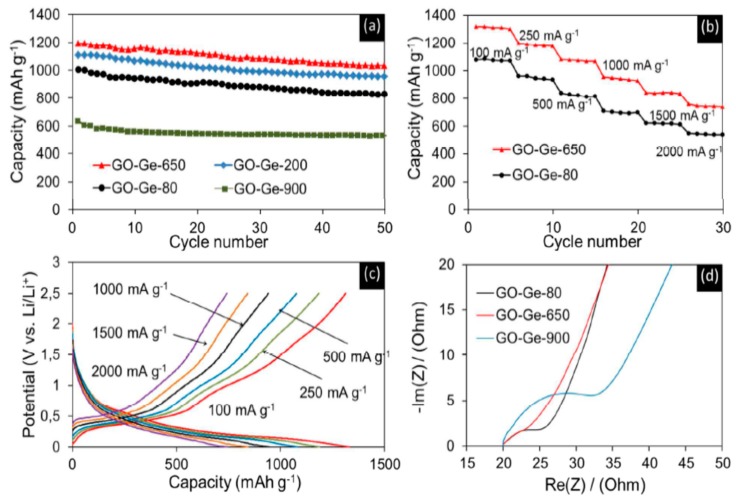
Evaluation of electrode behavior: (**a**) Charge capacities of GO–Ge-80, GO–Ge- 200 GO–Ge-650, and GO–Ge-900 upon repeated cycling. The cycles were steered at a rate of 250 mAg^−1^ among 0 V and 2.5 V vs. Li/Li^+^ (excluding GO–Ge-900 steered at 100 mA/g). (**b**) Charge capacities of GO–Ge-80 and GO–Ge-650 at diverse rates. (**c**) The charging-discharging curves for GO–Ge-650 composite for various current rates. (**d**) Nyquist plots of GO–Ge-80, GO–Ge-650, and GO–Ge-900 (Alexander et al. 2017) [168].

Graphene oxide (GO) reinforced by germanium oxide and lithium displays better processability. It was found that the charge capacity is a function of processing temperature: For temperatures up to 650 °C (Figure 12), an elevated charge capacity was detected. In the case of GO–Ge composite, the larger crystalline germanium grains attached over the electrode improved their performance when treated at 900 °C [168]. This can be quantified due to modified upgraded electrical conductivity observed at a higher temperature in the case of graphene-based composite materials. The GO–Ge-650 electrode material’s cycle achievement is good, with 250 mAg^−1^ storage capacity even after 50 cycles (Figure 12a). Thus, the GO–Ge-650 electrode active material’s rate performance is good, with a 2000 mAg^−1^ current rate for about 740 mAh g^−1^ capacity (Figure 12b). This corresponds to a 20-min charge-discharge period. The close contact among the graphene and the germanium moieties affords the material increased capacity at a higher current rate. In Figure 12d, Nyquist plots of GO–Ge-80, GO–Ge-650, and GO–Ge-900 EIS studies are displayed. For all three electrodes, the Nyquist plots showed skewed semicircles opening with the electrolyte solution resistance (standardized to 20), but it is interrupted by almost linear curves before the semicircle is complete. The semicircle in the Nyquist plots can be attributed to the lithium ion migration through the electrolytic surface interface and the charge transfer resistance, which occurs at an intermediate frequency in this case. The slope of the line (>45°) shows an amalgamation of electrolyte diffusion (Warburg impedance) and lesser fluctuations in the Li film because of lithiation at lower frequencies. In the case of GO–Ge-650 anodes, the higher load bearing capability corresponded to the smallest Li-ion propagation.

**Figure 13:**
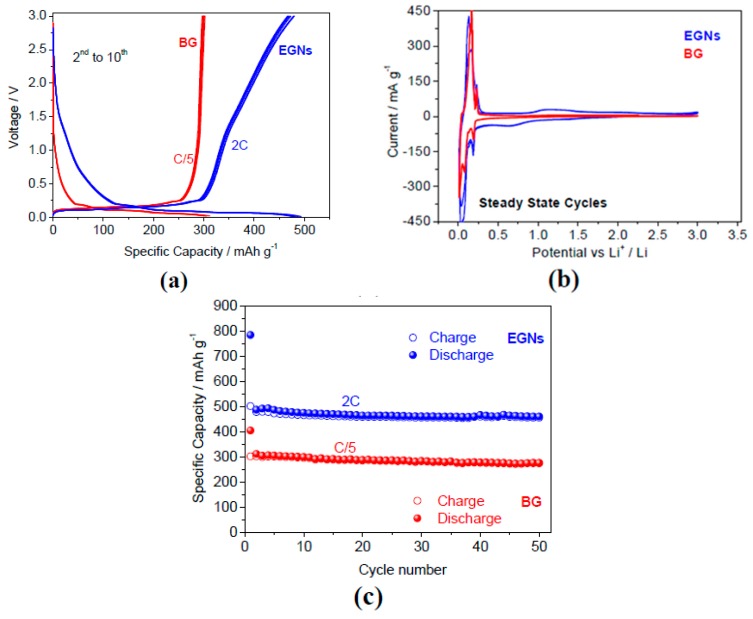
Galvanostatic voltage profiles from 2nd to 10th cycle (**a**), comparison of cyclic voltammetry at the steady state (**b**), and of the galvanostatic cycling performance (**c**) between the exfoliated graphite/graphene nanosheets (EGNs, blue line) and the blank graphite (BG, red line). Voltage range 0.01 V–3 V. Galvanostatic current density in lithium half cell 474 μA cm^−2^ (corresponding to 2C for the EGNS and C/5 for the BG). The scan rate of the cyclic voltammetry in three electrodes lithium cell 0.05 mV s^−1^. Electrolyte LP30 (EC:DMC 1:1 *v*/*v*, LiPF6 1M) [175].

Graphene is an attractive carbonaceous material consisting of carbon atoms arranged in a hexagonal structure, which has added advantageous properties for use as an anode, such as being an excellent conductor of electricity and heat, having the high specific surface area, high diffusivity, strong mechanical strength with flexibility, and outstanding chemical stability. The high surface area, greater electrical conductivity and high diffusivity of graphene allow increased ionic transport for intercalation and thus higher power [169,170,171,172]. The theoretical capacity of a graphene anode is higher than a graphite anode and is in the range of 744 mAh g^−1^ to 1448 mAh g^−1^ depending on the graphene morphology. Challenging problems observed in graphene anode in LIB are high operating voltage, high initial irreversible capacity, low loading, low coulombic efficiency, low performance at high current densities, and poor cycle life [173,174]. The development of LIBs with graphene anodes is promoted by using graphene in various forms, such as thin, flexible, transparent graphene sheets. To enhance the electrochemical properties of the graphene sheet, various approaches have been put forward to develop a low-cost scalable method for large-scale production. Exfoliated graphite/graphene nanosheets (EGNs) obtained by ultrasonication and ultracentrifugation of graphite-based solution exhibited a capacity of about 500 mAh g^−1^, which is higher than that observed in blank graphite (BG) (300 mAh g^−1^) [175]. In EGNs, the graphene could accommodate lithium in two sides of the carbon layer and the electrochemical reaction is Li + 3C ↔ LiC_3_, with a theoretical specific capacity of 744 mAh g^−1^, which is higher than the theoretical specific capacity of a graphite anode (372 mAh g^−1^). The EGNs show an additional Li-insertion slope, which extends from 0.25 V to 3.00 V that was absent in blank graphite (Figure 13a). The galvanostatic cycling performance confirms the additional Li^+^ uptake in the graphene phase of the carbon material that was evidenced in the region between 0.9 V and 2.5 V (Figure 13b), having similar stability. It was proposed that the Li intercalation in EGNs involves a hybrid mechanism within the graphene nanosheets. Figure 13c shows the long-term cycling properties of the graphene nanocomposite.

**Figure 14:**
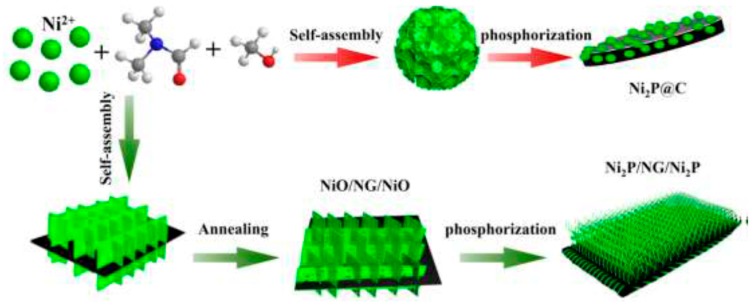
Schematic illustration of the synthesis of sandwich-like Ni2P/NG/Ni2P nanoarchitecture [182].

Some metals and metal oxides exhibit high theoretical gravimetric and volumetric capacities [176,177,178]. The drawback observed in these metals and metal oxides is low rate capability due to low electronic conductivity and poor cycling stability due to extreme volume expansion during cycling. These problems could be resolved by constructing hybrid materials with sandwiched architecture with highly conducting graphene and metal or metal oxide particles in layers to decrease the polarization effect and increase the electron/ion transfer kinetics [179,180,181]. The sandwich-like Ni_2_P nanoarray/nitrogen-doped graphene/Ni_2_P nanoarray nanoarchitecture (Ni_2_P/NG/Ni_2_P) electrode constructed by involving solvation method followed by annealing and phosphorization treatment is shown in Figure 14 [182].

This Ni_2_P/NG/Ni_2_P anode exhibited a capacity of 417 mAh g^−1^ at a current density of 300 mA g^−1^ after 100 cycles. The volume expansion during discharging could be stabilized by the hybrid structure through the interaction between Ni_2_P and the graphene matrix incorporated with a nitrogen dopant that leads to higher cycling stability. 

**Figure 15:**
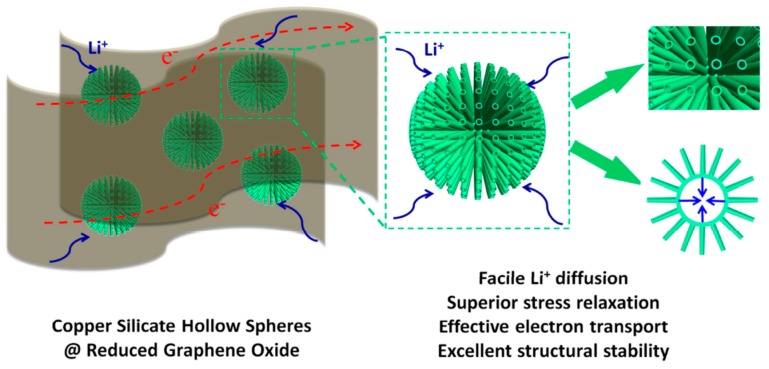
Schematic illustration of the copper silicate hydrate (CSH) hollow spheres encapsulated in reduced graphene oxide (RGO) (CSH/RGO) composite with facile Li^+^ diffusion [183].

The anode metal with the highest theoretical capacity was recognized as silicon, but it exhibited large volume change during Li insertion and extraction, which causes sudden capacity fading. Wei et al. [183] constructed a hierarchical hollow structure, such as hierarchical copper silicate hydrate (CSH) hollow spheres encapsulated in reduced graphene oxide (RGO) (CSH/RGO composite) using silicates, copper precursor, and graphene, which can withstand the volume change during Li insertion and extraction, thereby improving the cycling performance. The CSH/RGO composite presented higher capacity, rate performance, and cycling stability than that of CSH and RGO. The author suggested that the synergistic effect of the hierarchical hollow structure and electrical conductivity of reduced graphene oxide provided excellent long-life electrochemical performance. The hierarchical hollow structure provides facile Li^+^ diffusion with high-stress relaxation, effective electron transport, and excellent stability (Figure 15). These factors increased the capacity to a value of 890 mAh/g after 200 cycles at 200 mA/g and a capacity of 429 mAh/g after 800 cycles at 1000 mA/g. However, there was still an irreversible capacity loss observed in the first cycle due to the formation of SEI film. Graphene/metal oxide composites with Co_3_O_4_, MnO_2_, NiO, Fe_3_O_4_, and TiO_2_ showed high reversible capacity, good rate performance, and long cycle life [184,185,186,187,188]. In these graphene-based composites, graphene acts as a buffer to maintain the volume changes occurring in metal oxides [189]. 

Another interesting anode material is a tin oxide-based nanostructure material having a high theoretical capacity of 780 mAh g^−1^. However, huge volume change leads to pulverization and drop in the conductivity and electrochemical reversibility. Several studies showed that the electrochemical performance of SnO_2_ could be improved by using SnO/graphene composites, in which the volume change in SnO_2_ could be controlled by self-restacking of graphene [190,191,192]. Xu et al. [193] improved the conductivity and stability of SnO_2_ by preparing a composite using fluorine and graphene. It is believed that fluorine can increase the conductivity and graphene can increase stability. The derived fluorine-doped tin oxide (FTO) nanocrystal and reduced graphene oxide (RGO) (FTO/RGO) composite exhibited much higher capacity than that of the individual SnO_2_, SnO_2_/reduced graphene oxide (SnO_2_/RGO), fluorine-doped tin oxide (FTO), and FTO/RGO composite, as given in Table 1. 

The specific capacity of FTO/RGO was much higher compared to that of its individual components. Comparison of charge transfer resistance (Rct) and contact resistance (Rf) of the SnO_2_, SnO_2_/RGO, FTO, and FTO/RGO samples confirmed that the Rf value of FTO/RGO was much lower compared to that of its individual components. The authors claimed that the thinner growth of the SEI layer at the electrode-electrolyte interface of the FTO/RGO samples reduced the contact resistance. 

Fast charging and discharging could be achieved by graphene doping [194]. For instance, N-doped graphene electrodes exhibited a very high capacity of 1043 mAh g^−1^ and B-doped graphene electrode exhibited 1549 mAh g^−1^ with higher coulombic efficiency and cycle performance in comparison with undoped graphene. The doped N-doped and B-doped graphene can be quickly charged and discharged for a very short time of 1 h to several tens of seconds, showing high rate capability and excellent long-term cyclability at the same time [169]. Niobium oxide (Nb_2_O_5_) is considered as another promising anode material in a lithium-ion battery for its advantage of preventing the formation of SEI at higher operation potentials. Also, the Nb_2_O_5_ materials exhibited high rate capability and excellent cycle durability [195]. However, these materials exhibited low ion diffusion and charge transfer [196]. Yan et al. [197] showed that Nb_2_O_5_ in a carbon matrix showed enhanced rate performance and cycle durability, especially in the oxygen-deficient Nb_2_O_5_, such as Nb_2_O_5−x_. The carbon matrix in Nb_2_O_5−x_ shielded the volume change during charging and discharge processes. Also, the carbon matrix increases the conductivity, which in turn enhances rate performance and cycle durability [197].

**Figure 16:**
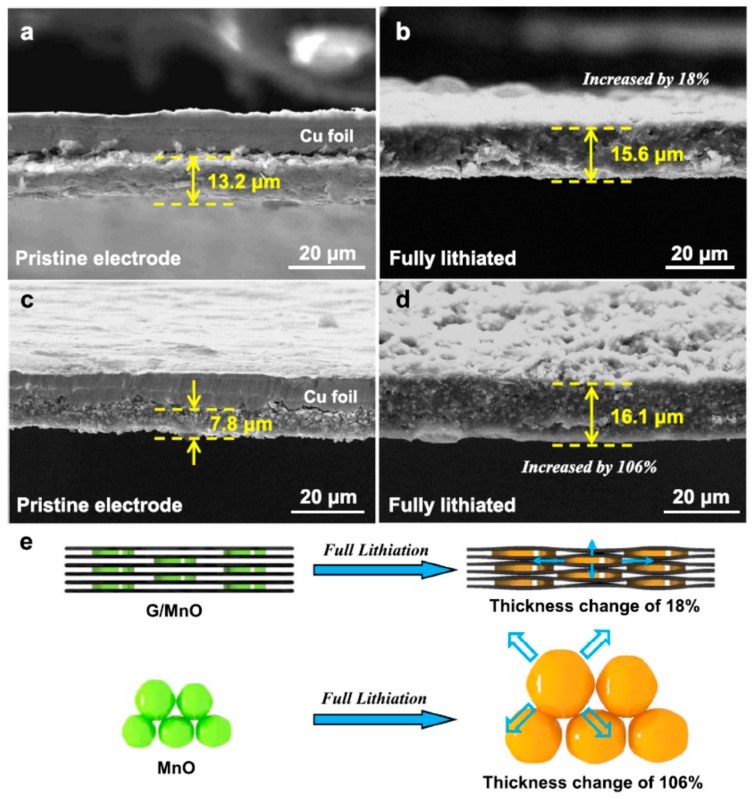
Cross-sectional SEM images of the G/MnO-800 and MnO electrodes: (**a**) G/MnO-800 and (**c**) MnO; Fully lithiated state: (**b**) G/MnO-800 and (**d**) MnO). (**e**) Schematic illustration of the thickness change for the G/MnO-800 and MnO during the full lithiation process [199].

Wu et al. [198] fabricated SnO_2_ quantum dots loaded on the sulfur-doped reduced graphene oxide that could retain their tiny sizes during Li insertion/extraction and thus reduce the volume expansion. Defects in the structure introduced by the sulphur dopant allow ease of charge transfer and Li^+^ ion diffusion. The specific capacity at 100 mAg^−1^ was 897 mAh g^−1^ and 88% of original capacity was maintained after 500 cycles at 500 mAg^−1^. Sheng et al. [199] have constructed MnO_2_ nanosheets with the sizes of 50–100 nm anchored between the graphene oxide sheets to form a densely stacked graphene/MnO (G/MnO) architecture with some micropores (0.4–0.8 nm) in the graphene sheet. The graphene confinements in G/MnO enhance the electrical conductivity and reduce the charge transfer impedance through fast Li^+^ ion diffusion and low charge transfer resistance. The capacity and rate capability derived from this graphene/MnO hybrid system are 1000 mAh g^−1^ at 0.1 Ag^−1^, and 604 mAh g^−1^ at 2 Ag^−1^, respectively, which is much higher than those reported for MnO (777 mAh g^−1^ at 0.1 Ag^−1^, 343 mAh g^−1^ at 2 Ag^−1^), graphene (648 mAh g^−1^ at 0.1 A g^−1^, 282 mA h g^−1^ at 2 Ag^−1^) and MnO/graphene (804 mAh g^−1^ at 0.1 Ag^−1^, 453 mAhg^−1^ at 2 A g^−1^) [200,201,202]. The volumetric capacity of G/MnO is 2.27 times higher than MnO/graphene and 5.40 times higher than graphene. G/MnO, MnO/graphene, and graphene exhibited volumetric capacity of 2288 mA h cm^−3^, 1008 mAh cm^−3^, and 424 mAh cm^−3^, respectively [199]. The higher capacity and rate capability are due to the densely stacked graphene/MnO (G/MnO) architecture with some micropores, which offers easy diffusion for ion transport. In addition, the cycle stability of G/MnO is much higher than MnO, graphene, and MnO/graphene. There is a change in thickness of the G/MnO-800 electrode of about 18%, which is lower than that of pure MnO (106%). Regarding the expansion/contraction during the charge-discharge process, MnO nanosheets preferentially expand and shrink and were maintained by confinement of the graphene sheets as illustrated by TEM imaging before and after lithiation (Figure 16). However, there is an inevitable initial capacity loss (initial Coulombic efficiency of 65%) due to electrolyte decomposition and formation of the SEI layer. A detailed study reported the structure evaluation by TEM analysis for CeO_2_/graphene composites during lithiation and delithiation processes, revealing that during the lithiation processes, Li_2_O layers were formed on the surfaces of the CeO_2_ and graphene sheets and most of these layers disappeared during delithiation. The leftover Li_2_O layers on CeO_2_ and the graphene sheets could form stable SEI layers, which leads to capacity loss [188]. 

**Figure 17:**
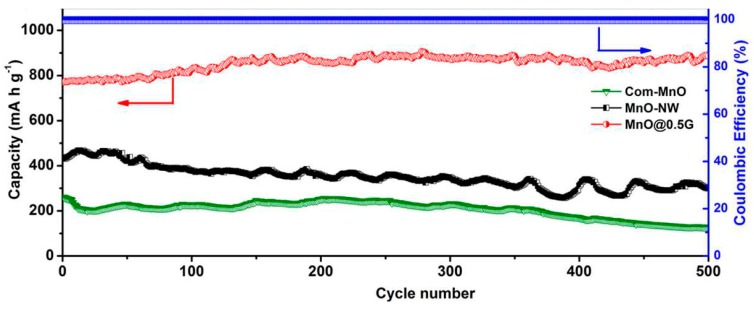
Cycling performance of MnO–graphene core-shell nanowires [203].

Xiao et al. [203] constructed MnO–graphene core-shell nanowires, in which graphene was coated on MnO, which exhibited comparable electrochemical performance as that of the densely stacked graphene/MnO (G/MnO) architecture constructed by Sheng et al. [199] with reversible capacities of 1185 mAh g^−1^ at 50 mAg^−1^, rate capability (884 mAh g^−1^ at 500 mAg^−1^ and 508 mAh g^−1^ at 3 A g^−1^), high initial coulombic efficiency (86.3 %), and long-term cyclic stability as long as 500 cycles at 1A g^−1^, 15% higher than the initial capacity (Figure 17) [203].

N-doped carbon (C_3_N) with excess nitrogen (C_2.67_N) is a promising material for use in LIBs with a theoretical capacity of 837.06 mAh g^−1^ [204]. The excess C in C_3_N (C_3.33_N) exhibited a high reversible capacity of 840.35 mAh g^−1^ with fast charging-discharging rate [205]. C_3_N/graphene has a theoretical capacity of 1079 mAh g^−1^ and exhibits good electric conductivity with high stability [206].

**Figure 18:**
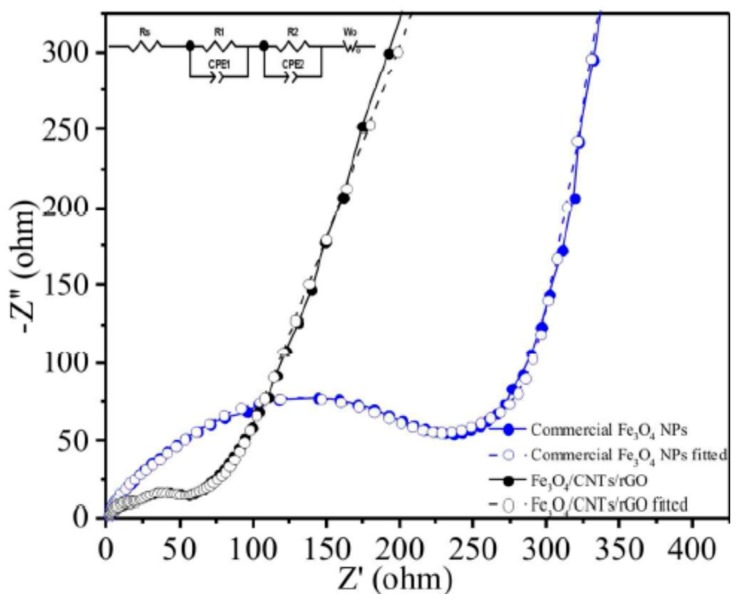
Electrochemical impedance spectrum (EIS) and fitting results of the Fe_3_O_4_/CNTs/rGO composite and commercial Fe_3_O_4_ NPs electrodes [209].

Magnetite (Fe_3_O_4_) has been considered as an anode material due to its abundance, low-cost, and eco-friendly characteristics. The theoretical capacity of Fe_3_O_4_ is 926 mAh g^−1^ and the electronic conductivity is 2 × 10^4^ Sm^−1^. However, huge volume changes during Li^+^ ions insertion/extraction processes were observed in Fe_3_O_4_, which leads to rapid capacity loss [207]. Zhou et al. [208] found that porous Fe_3_O@C nanospheres performed as a high-performance anode for a lithium-ion battery. Yang et al. [209] constructed nanoparticles of Fe_3_O_4_ interconnected by carbon nanotubes that were sandwiched by rGO layers to form a network (Fe_3_O_4_/CNTs/rGO) by a facile route without any binders. This composite network exhibited a reversible capacity of 540 mAh g^−1^ at a very high current density of 10 A g^−1^, and a stable capacity of 1080 mAh g^−1^ after 450 cycles at 1 A g^−1^). The faster transportation of Li^+^ ions and electrons in the Fe_3_O_4_/CNTs/rGO composite network resulted in superior rate performance as evidenced from the electrochemical impedance spectrum (EIS) of the commercial Fe_3_O_4_ NPs and Fe_3_O_4_/CNTs/rGO electrodes after the first cycle (Figure 18). The resistance values of the Fe_3_O_4_/CNTs/rGO composite electrode are lesser than that of the commercial Fe_3_O_4_ NPs electrode. 

Fe_3_O_4_ nanoparticles/graphene foam with a hierarchical structure with well-dispersed Fe_3_O_4_ nanoparticles on a graphene matrix exhibited a capacity of 1198 mAh g^−1^, which was higher than that of theoretical capacity value for Fe_3_O_4_ (926 mAh g^−1^) and pure GF (300 mAh g^−1^) after 400 cycles [210]. The network-structured graphene minimizes the direct contact with positively-charged Fe_3_O_4_ NPs that will control the aggregation of the NPs during charge-discharge processes. The hierarchical structure enhances electron/ion transport.

**Figure 19:**
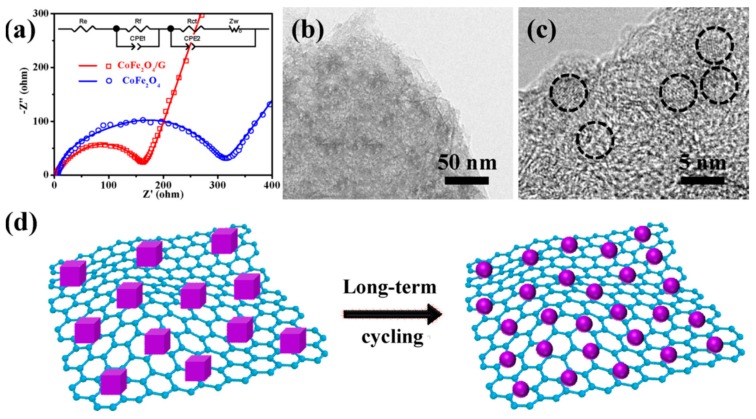
Nyquist plots of the CoFe_2_O_4_/G and CoFe_2_O_4_ electrodes (hollow symbols: measured data; continuous lines: fitting curves) (**a**); TEM image (**b**) and HRTEM image (**c**) of the CoFe_2_O_4_/G electrode after 200 cycles at 1 A g^−1^; The schematic illustration of the morphological and structural evolution of the CoFe_2_O_4_/G electrode (**d**) [212].

The theoretical capacity of a Co_3_O_4_ anode in LIB is 890 mAh g^−1^, involving eight electrons, and is much higher than that graphite, but is limited by its poor cycling stability resulting from huge volume change, as with other oxide anodes [211]. Yang et al. [212] constructed a Co_3_O_4_/graphene sandwich-like hybrid electrode by direct electrophoretic deposition without using any binder. The Co_3_O_4_/G hybrid electrode showed initial discharge and charge capacities of 1303.9 and 946.0 mAh g^−1^, respectively, with initial Coulombic efficiency (CE) of 72.6% (Figure 17). The reversible discharge capacity of Co_3_O_4_/G hybrid electrode was 1113 mAh g^−1^ (100 cycles), which is much higher than that of Co_3_O_4_ electrode (583 mAh g^−1^ after 50 cycles). The higher electrochemical performance of the Co_3_O_4_/G hybrid electrode is due to: (i) Many voids formed by Co_3_O_4_ nanocubes embedded between graphene sheets, which can provide buffer space for huge volume expansion during material during cycling; (ii) improved electronic conductivity by flexible graphene; and (iii) higher energy density and less agglomeration as there are no binders or conductive additives. The nanocubes of CoFe_2_O_4_/graphene composite (CoFe_2_O_4_/G electrode) also exhibited comparable cycling performance and rate capability to that of the Co_3_O_4_/G hybrid electrode. The cycling performance of the CoFe_2_O_4_/G electrode was 1109 mAh g^−1^ after 100 cycles at 0.2 Ag^−1^ and 835 mAh g^−1^ after 200 cycles at 1 Ag^−1^ and rate capability was 420 mAh g^−1^ at 5 A g^−1^. The CoFe_2_O_4_/G electrode displayed better diffusion behavior of lithium ions than CoFe_2_O_4_ electrodes. The overall shape of the electrode material CoFe_2_O_4_/G electrode was well retained, as evidenced by TEM and HRTEM images of the CoFe_2_O_4_/G electrode after 200 cycles at 1 Ag^−1^ (Figure 19) [212]. 

**Figure 20:**
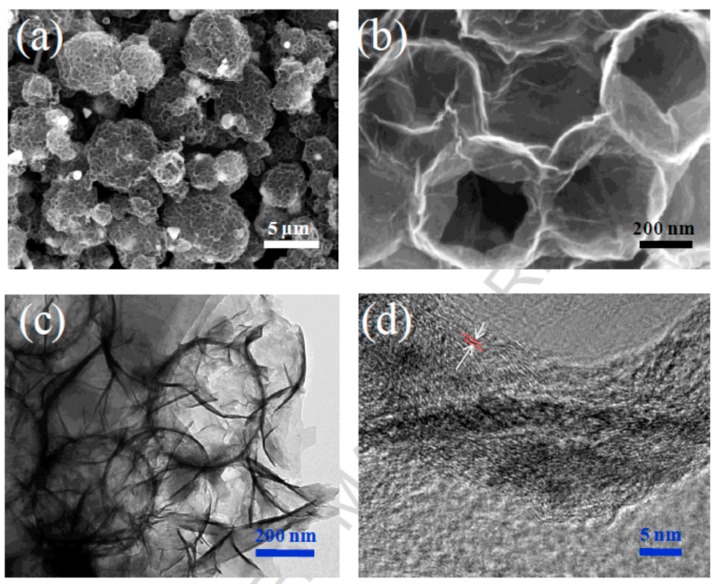
Morphologies of the 3DGPM: (**a**, **b**) SEM images; (**c**, **d**) TEM and HRTEM images [221].

Comparison of two-dimensional (2D) graphene and 3D porous graphene showed that in 2D-graphene, there are strong van der Waals interactions between the nanosheets, which could hinder the reversible capacity [213,214,215,216,217,218]. However, in 3D porous graphene, there are defects and interconnected pores, which offer easy diffusion for ion transport and relief of volume expansion during the Li^+^ ions intercalation/deintercalation process, thereby improving the capacity, rate capability, and stability [213,215,219]. Chemical etching and templating [218,219,220] are commonly used methods to prepare graphene with porous architecture. Zhu et al. [221] fabricated a three-dimensional porous graphene microspheres (3DGPM) anode with interconnected hollow pore structure by a template-sacrificing method. The 3DGPM has specific hollow micro-spherical structure. The presence of graphene spheres (diameter of 1–10 μm) with interlinked hollow spherical embossed structure was confirmed by scanning electron microscopy. Hollow spherical graphene shells dispersed on graphene microspheres was confirmed by transmission electron microscopy. The multilayer reduced graphene on the boundary of graphene shells was presented by high-resolution TEM (Figure 20). 

**Figure 21:**
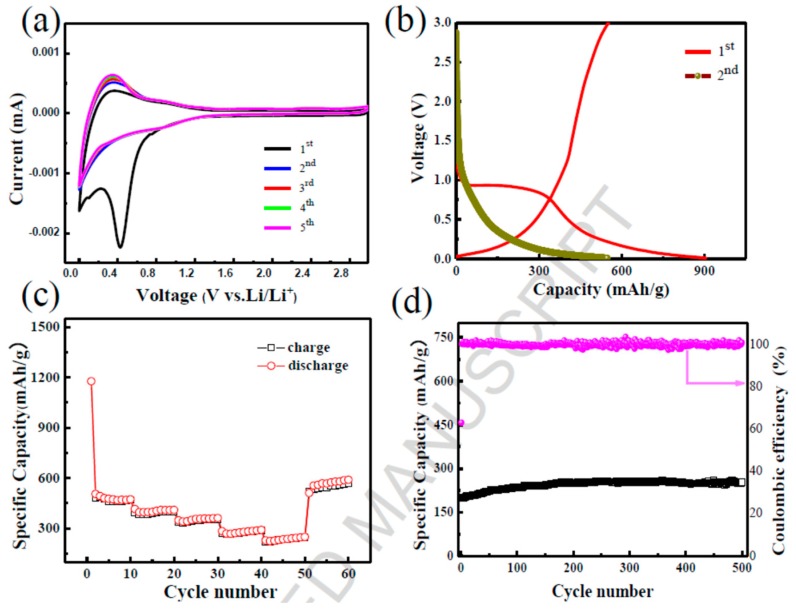
Quantitative analysis of lithium storage behavior of the 3DGPM. (**a**) CV curves; (**b**) Charge and discharge curves of the 3DGPM at 0.1 C; (**c**) rate capability at various current density; (**d**) capacity retention at 2 A g^−1^. [221].

The 3DGPM exhibited a reversible capacity of 551.4 mAh g^−1^ (at 100 mA g^−1^) and rate capability of 245.8 mAh g^−1^ (at 2 A g^−1^). The first discharge–charge capacities were 851.1 and 402.4 mAg^−1^ (at 0.1 A g^−1^). The 500th discharge capacity was 245.8 mAh g^−1^ (at 2 A g^−1^). The charge transfer resistance (R1) of 3DGPM was 82.5 Ω, which was much lower than that of the RGO (258.3 Ω) (Figure 21). The presence of specific hollow micro-spherical structure provides pathways for fast electron transfer during repeated cycling and offer voids for volume expansion [221].

**Figure 22:**
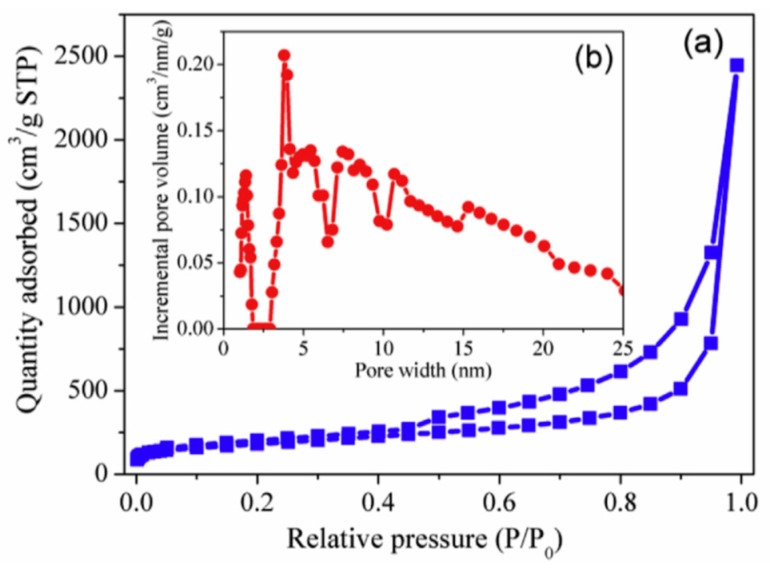
Nitrogen adsorption-desorption isotherm (**a**) and pore size distribution (**b**) of coal-based porous graphene (CPG) [222].

Xing et al. [222] adopted graphitization of anthracite, followed by a modified Hummers method, and then rapid reduction at high temperature. This method resulted in continuous nanosheets with a hierarchical porous structure having micro/meso/macropores, with high specific surface area and large pore volume with enormous structural defects with a wide pore size distribution ranging from 1.0 to 25.1 nm (Figure 22). 

These porous graphene materials exhibited lower capacity compared to metal oxide-doped graphene but have higher cycling performance. The capacity and rate capability are 770 mAh g^−1^ (at 0.1 C), and 274 mAh g^−1^ (10 C) and 224 mAh g^−1^ (20 C), respectively. The cycling performance reached up to 98.0% (after 110 cycles), which is higher than that of a graphene anode (95.7%), with near 100% efficiency was reported. The graphene orientation results in different properties in such a way that vertically-oriented graphene nanowalls (VGNWs) have three-dimensional network morphology with long and thin edges having a large specific surface area, which provides good electrical properties as well as structural stability [223]. Chemical vapor deposition (CVD) and plasma-enhanced chemical vapor deposition (PECVD) are used to synthesize vertically-oriented graphene nanowalls (VGNWs). Faster deposition growth at a lower temperature could be achieved by PECVD [224]. Yang et al. applied a plasma-based approach to fabricate VGNWs with tailored properties by adjusting the experimental conditions such as argon flow rate, growth temperature, RF power, and deposition times [225]. Under optimum conditions, the specific discharge capacity reaches 400 mAh g^−1^ in the first charge-discharge cycle and was lower than the graphene sheets prepared by thermally exfoliation of graphene oxide at 1050 °C [226], which have the specific capacity of 672 mAh g^−1^ at a current density of 0.2 mAcm^−2^ and thermally reduced graphite oxide at 1050 °C, which has the specific capacity of 835 mAh g^−1^ at a current density of 50 mA g^−1^ [227]. 

More than 20 years ago, the carbon anode enabled the Li-ion battery to become commercially viable and is still the material of choice for the anode. The electrochemical activity of carbon is the result of Li intercalation between the graphene planes offering good mechanical 2D stability, electrical conductivity, and Li transport. In this way, it is possible to store up to one Li atom per six C. A summary of the application of carbon anodes Li-ion batteries is presented in Table 2.

## 3. Conclusions and Future Directions 

In this review, we have reviewed the utilization of carbon-based nanomaterials as potential anode materials for Li-ion batteries. Based on the literature survey, it was observed that the carbon-based nanomaterials are promising materials which can efficiently be used as anode materials for Li-ion battery applications. Various carbon-based materials, such as carbon nanotubes, carbon nanofibers, carbon xerogels, carbon nanosprings, and graphene, were used as efficient Li-ion battery anode materials. Although carbon-based nanomaterials are efficiently used as anode materials for Li-ion battery applications, to fulfill the need for high power electrochemical cells, there is a need to utilize higher surface area materials which should inherently have a higher reaction rate. The natural step is to look at nanomaterials with surface areas more than 5 m^2^ g^−1^ and up to several hundred m^2^ g^−1^. However, the increase in surface area will also increase the side-reactions and potentially reduce the safety of the cells. In addition, the nanomaterials in batteries show low density. Therefore, not all materials are appropriate to use at the nanolevel, just those that undergo the desired reaction in a highly selective manner. New material development for low-cost anodes is expected to endow both small and large batteries with superior rate capability, safety, and cycle performance. 

## Figures and Tables

**Table 1:** Capacities of SnO_2_, SnO_2_/RGO, FTO, and FTO/RGO during charge and discharge processes at a current density of 100 mAg^−1^ [193].

Sample	SnO_2_	SnO_2_/RGO	FTO	FTO/RGO
1st discharge capacity (mAh g^−^^1^)	787	1536	1168	2108
200th discharge capacity (mAh g^−^^1^)	201	821	265	1439
Capacity retention after 200th	26%	53%	23%	68%

**Table 2:** A summary of the applications of carbon anodes in Li-ion materials.

Materials	Significant Applications/Findings observed	Ref.
ZnCo_2_O_4_/ rGO	The electrochemical properties of ZnCO_2_O_4_/rGO composite material exhibited a raised cycling capability as high as 1613 mAh g^−1^ at 500 mA g^−1^ over 400 cycles	[228]
Sn@ G	The 3D hybrid Sn@Graphene anode displays an elevated cycling capability of 1022 mAh/g at 0.2 C and 270 mAh/g at 10 C along with tremendously longer cycling stability at higher rates, such as 682 mAh/g cycling stability at 2 A/g. This is roughly about 96.3% even after 1000 cycles, which is perhaps the best rate capacity and lengthiest cycle life ever testified for Sn-based battery anode for Li ions.	[229]
Ga_2_O_3_/rGO	The Ga_2_O_3_/rGO nanocomposite has been proven to be a promising electrode material for use as a Li-ion anode with superior performance. This enhancement in performance is due to the electron transport characteristics created due to the presence of controlled open porous, crystal structure, and crystal morphology by doping with rGO.	[230]
GeO/GO	A test carried to evaluate the performance of different materials for lithium ion battery anodes revealed GO containing only 51% wt germanium to exhibit good storage capacity > 1000 mAh g^−1^ at 250 mA g^−1^.	[168]
Graphene-like graphite (GLG)	GLG’s discharge capacity above 1 V increases the oxygen percentage, with successive discharge capacity reaching 673 mAh g^−1^. Thus, GLG can act as an ideal anode for Li-ion storage cells.	[231]
Co_3_Sn_2_@ Co-NG	The Co_3_Sn_2_@Co-NG hybrid nanocomposite shows an unexpected reversible capacity of 1615 mAh/g at 250 mA/g after 100 cycles when used as an anode. These Co_3_Sn_2_@Co-NG composites were able to produce 100% coulombic efficiency.	[232]
CuMn_2_O_4_/G	These nanostructured CuMn_2_O_4_/graphene composites were able to deliver a larger capacity of 935 mAh/g after 150 cycles, corresponding to a current density of 50 mA/g. Additionally, it was further confirmed that CuMn_2_O_4_ composite changes into a nanosized CuO–MnO composite material with a spongy porous structure during discharge–charge processes.	[233]
ZnO/rGO	ZnO/rGO as an anode material in a lithium-ion battery exhibits brilliant cycling stability and improved charge capacity rates, because of its doughnut-like ZnO assembly. ZnO/rGO nanocomposite provides high electrical conductivity due to its short diffusion length.	[234]
Sn/SnO_2_@Graphene	Improved initial capacity as high as 2970.3 mAh g^−1^ makes Sn/SnO_2_@Graphene composites an ideal candidate for high-performance electrodes for Li/Na-ion batteries.	[235]
Co_3_O_4_/rGO	Co_3_O_4_/rGO composites are preferred materials for lithium ion batteries due to their superior electrochemical behavior as an anode material. This superior behaviour is as a result of the steady changes in morphology that occur with respect to thicknesses of the material during the process of cycling, thereby reducing the stress caused during cycling by providing a smaller diffusion path length for Li-ions and electrons.	[236]
3DG/Fe_2_O_3_	The 3DG/Fe_2_O_3_-based aerogel, when used as anode material for lithium-ion battery, displayed an extremely large storage capacity of 1129 mAh/g at 0.2 A/g after 130 cycles. This highly flexible anode retained a storage capacity as high as 98% after 1200 cycles at 5 A/g.	[237]
Graphene-Si aerogels	Electrochemical investigations indicated that the fabricated graphene–Si aerogels were capable of delivering 104% charging capacity at 0.8 A/g. Carbon nanotubes functionalized with silicon were utilized to achieve capacities as high as 1415 mAh/g at a discharge rate of 0.05 C. Thus, it is expected that graphene–Si aerogels will play a noteworthy role in lithium ion batteries.	[238]
Graphene nanoflakes ink	By optimizing the cell composition, the best battery behaviour of 165 mAh g^−1^ was achieved, corresponding to an energy density as high as 190 Wh kg^−1^ for an average of 80 cycles.	[74]
TiO_2_-B-G	TiO_2_-B-graphene nanocomposite material doped with 18.8 wt% of graphene exhibits promising electrochemical characteristics with charge capacities reaching 264 and 255 mA hg^−1^ at a current rate of 1 C. The storage capacity is as high as 171 mAh g^−1^ and was retained after 100 cycles at 5 C. The increase in graphene concentration above 27.7 wt%, diminished the lithium storage properties due to the accumulation of graphene structures.	[239]
Fe_2_N@AC@rGO	The amorphous carbon (AC) shell and graphene oxide (rGO) network were able to provide long-term cyclic stability along with extraordinary rate capability of 303 mAh g^−1^ at 10 A g^−1^.	[240]
